# The Cytokine Network in Colorectal Cancer: Implications for New Treatment Strategies

**DOI:** 10.3390/cells12010138

**Published:** 2022-12-29

**Authors:** Heidi Braumüller, Bernhard Mauerer, Johanna Andris, Christopher Berlin, Thomas Wieder, Rebecca Kesselring

**Affiliations:** 1 Department of General and Visceral Surgery, Medical Center, Faculty of Medicine, University of Freiburg, 79106 Freiburg, Germany; 2 Department of Vegetative and Clinical Physiology, Institute of Physiology, Eberhard Karls University Tübingen, 72074 Tübingen, Germany

**Keywords:** interferon, interleukin, tumor necrosis factor, tumor microenvironment, tumor progression, tumor surveillance, consensus molecular subtypes

## Abstract

Colorectal cancer (CRC) is one of the most frequent tumor entities worldwide with only limited therapeutic options. CRC is not only a genetic disease with several mutations in specific oncogenes and/or tumor suppressor genes such as APC, KRAS, PIC3CA, BRAF, SMAD4 or TP53 but also a multifactorial disease including environmental factors. Cancer cells communicate with their environment mostly via soluble factors such as cytokines, chemokines or growth factors to generate a favorable tumor microenvironment (TME). The TME, a heterogeneous population of differentiated and progenitor cells, plays a critical role in regulating tumor development, growth, invasion, metastasis and therapy resistance. In this context, cytokines from cancer cells and cells of the TME influence each other, eliciting an inflammatory milieu that can either enhance or suppress tumor growth and metastasis. Additionally, several lines of evidence exist that the composition of the microbiota regulates inflammatory processes, controlled by cytokine secretion, that play a role in carcinogenesis and tumor progression. In this review, we discuss the cytokine networks between cancer cells and the TME and microbiome in colorectal cancer and the related treatment strategies, with the goal to discuss cytokine-mediated strategies that could overcome the common therapeutic resistance of CRC tumors.

## 1. Introduction

### 1.1. Classical Classification of CRC

In 2020, CRC was the third most common cancer entity and the second most deadly cancer worldwide [1]. CRC accounted for approximately 10% of all cancers and 9.4% of all cancer deaths [1]. The main risk factors are environmental factors such as western diet, western lifestyle or physical inactivity, leading to the highest estimated number of new cases in China and the United States. Due to the risk factors and increasing number of CRC cases, the incidence to develop CRC in younger years is rising [2,3].

CRCs are a very heterogeneous group of diseases with several mutations and epigenetic aberrations. Approximately 5% of all CRC cases are inherited, caused by mutations in some CRC-typical genes [4]. The most frequent syndromes for hereditary CRC are the Lynch syndrome (hereditary nonpolyposis colorectal cancer) and familial adenomatous polyposis [5,6]. Approximately 20 % of all CRC cases are familial with a positive family history but without the classical inherited mutations that classify the inherited cancer variants [7].

Approximately 75% of all CRCs are sporadic with specific mutations along the way from adenomas to carcinomas [8].

The genomic instability pathway is one component of this transforming progress. Chromosomal instability (CIN) is characterized by the frequency of loss of heterozygosity (LOH) and aneuploidy and accounts for 70% of all sporadic CRCs. In addition to this chromosomal rearrangement, mutations in several genes or loss of genes occur. The first mutation occurs in a tumor suppressor gene, the adenomatous polyposis coli (APC) gene. The APC protein is part of the WNT pathway, regulating differentiation, adhesion, apoptosis, migration and chromosomal segregation [9]. The APC mutation is followed by the activating mutation of the oncogenes Kirsten rat sarcoma virus oncogene (KRAS) [10], the proto-oncogene B-Raf and v-Raf murine sarcoma viral oncogene homolog B (BRAF) [11] and the phosphatidylinositol-4,5-bisphosphate 3-kinase, catalytic subunit alpha (PIK3CA) [12], all leading to a constant activation of the mitogen-activated protein (MAP) kinase pathway and the phosphatidylinositol 3-kinase (PI3K) pathway. Both signaling pathways increase cell survival and proliferation in CRC [13,14]. Next, chromosome 18q21 is often deleted with the tumor suppressor genes SMAD2, SMAD4 and deleted in colorectal carcinoma (DCC). Both SMAD genes are members of the transforming growth factor-beta (TGF-*β*)-signaling pathway, facilitating the TGF-*β*-induced cell cycle arrest [15]. DCC encodes for a transmembrane receptor for netrin-1 acting as a tumor suppressor gene [16]. Finally, a mutation in the tumor suppressor gene TP53 occurs, encoding for the tumor suppressor p53, whose inactivation is a late event in the CRC carcinogenesis process [17]. A non-functional p53 protein promotes cell proliferation, migration and invasion [18].

The second molecular pathway of colorectal cancer is the microsatellite instability (MSI) pathway. Microsatellites are short DNA tandem repeats (two to five base-pair repeats) spread across the whole genome. MSI is caused by dysfunction of the DNA mismatch repair (MMR) during DNA replication. The MMR complex identifies base-pair mismatches or insertion-deletion loops that are caused by DNA damage or inaccurate DNA polymerase transcription and repairs them. MSI is the result of the MMR inability to repair the errors. The MMR complex consists of at least seven proteins and the corresponding genes. Five of the genes are known to be mutated in MSI CRC: MutL Homolog 1 (MLH1), MLH2, MutS Homolog 6 (MSH6), Protein Homolog 1 (PMS1) and PMS2 [19,20]. Microsatellite instability accounts for approximately 15 % of all sporadic CRCs.

**Figure 1:**
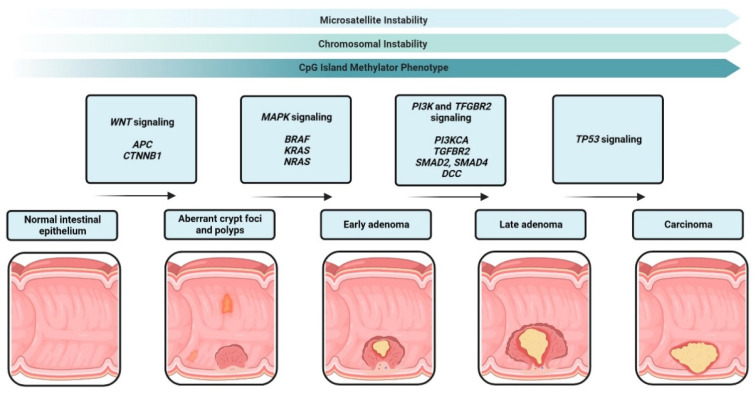
Adenoma-carcinoma sequence from healthy epithelium to carcinoma in situ. Carcinogenesis progresses through the accumulation of several mutations in oncogenes such as BRAF, KRAS or HRAS and tumor suppressor genes such as APC, SMAD2 or TP53.

The third molecular pathway of colorectal cancer is the CpG island methylator phenotype (CIMP). CIMP is an epigenetic instability leading to hypermethylation in the promotor region of several genes. Genes with hypermethylated promotors are silenced and several tumor suppressor genes, such as MLH1 or APC, mutated in colorectal cancer (MCC), and others are not transcribed any more [21]. In CRC and several other tumors, epigenetics and genetics cooperate and play an important role in tumor progression [22]. Figure 1 summarizes the sequence from healthy tissue to carcinoma in situ.

As there is great heterogeneity within each molecular pathway and sporadic CRCs show features of multiple pathways, the classification with only the CIN, MSI and CIMP phenotypes had to be extended:

(I) The conventional pathway that harbors CRCs that are CIMP^-^, CIN^+++^ and MSS/MSI^low^.
(II) The serrated pathway that contains two groups.
  - Cancer cells that are MSS with a mutated KRAS and a low CIMP phenotype.
  - MSS cancers with BRAF mutations and a high CIMP phenotype.
(III) The MSI pathway, consisting of two groups.
  - MSI, CIMP-high tumors with frequent BRAF mutations and MLH1 methylation.
  - MSI, CIMP-negative tumors with KRAS mutations and no BRAF mutations.

### 1.2. The Consensus Molecular Subtype Classification

**Figure 2:**
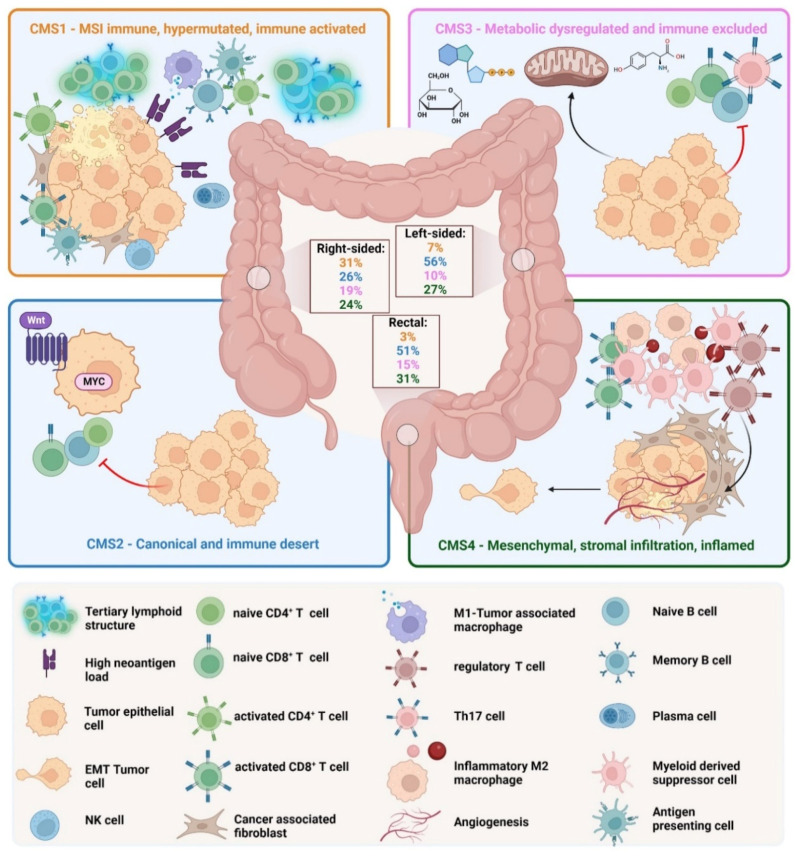
Consensus molecular subtypes of CRC. CMS1, the MSI immune subtype, is characterized by infiltration of lymphocytes and the formation of tertiary lymphoid structures. CMS2, the canonical subtype, is characterized by absent immune infiltration. CMS3, the metabolic subtype, is characterized by immune cell exclusion. CMS4, the mesenchymal subtype, is characterized by the infiltration and activation of mesenchymal cells such as fibroblasts.

Although the classical classification provides good information about the specific morphology and molecular alterations, it does not provide prognostic or clinical information. Tumor progression and therapeutic options depend not only on the genetic and epigenetic phenotype of the cancer cells but also on the complex tumor microenvironment. To stratify the treatment strategy and integrate molecular and histologic features of CRCs, an international consortium analyzed a large cohort of primary CRCs and established a new molecular classification [23]. In 2015, the consortium classified CRC into four consensus molecular subtypes (CMS) based on the bulk transcriptomic sequencing (Figure 2).

(I) CMS1 is the so-called MSI-like subtype, characterized by MSI and a hypermutated profile with mutations in the MLH1 and BRAF genes. CMS1 patients have a diffuse immune infiltrate of T helper 1 (T_H_1) cells, natural killer (NK) cells and cytotoxic T lymphocytes (CTL). This subtype shows also a CIMP phenotype. Approximately 14% of CRCs belong to the CMS1 subtype.
(II) CMS2 is the so-called canonical subtype and includes CRCs with higher CIN and a high level of somatic copy number alterations. CMS2 CRCs show an upregulated WNT and MYC pathway and no dendritic cell (DC) recruitment. The CMS2 subtype is only poorly immunogenic with no immune infiltrate and no immune regulatory cytokines. Approximately 37% of CRCs belong to CMS2.
(III) The CMS3 subtype is the metabolic subtype, characterized by the dysregulation of the glucose pentose, nitrogen, fatty acid and several other metabolic pathways. CMS3 shows CIN and CIMP^low^ status. The level of KRAS mutations is higher compared with other CMS phenotypes. CMS3 is, similar to CMS2, only poorly immunogenic with no immune infiltrate and no immune regulatory cytokines. Approximately 13% belong to the CMS3 subtype.
(IV) CMS4 is the mesenchymal phenotype with high CIN and a strong expression of the epithelial–mesenchymal transition (EMT). CMS4 tumors show TGF-*β* signaling and a high C-X-C Chemokine Ligand 12 (CXCL-12) expression. The CMS4 phenotype shows high levels of infiltrating CTLs, macrophages and stromal cells [24]. Approximately 23% of CRC tumors fall into CMS4. Although CMS4 patients show high levels of leukocyte infiltration, patients with CMS4 tumors have the worst prognosis of the four subtypes [23].

The CMS classification with striking differences in overall survival and treatment opportunities between the subtypes made clear that CRC is not only a genetic and epigenetic disease but also that the microenvironment plays a major role in cancer initiation, cancer progression and invasion. CRCs are comprised of cancer cells and non-malignant cells including tumor-infiltrating lymphocytes, cancer-associated fibroblasts (CAFs), cancer-associated macrophages, endothelial cells and the extracellular matrix. In colorectal cancer, tumor progression and invasion depend on infiltrating T cells and interferon-γ (IFN-γ) signatures [25]. In CRC, the presence of specific tumor-infiltrating lymphocytes (TIL) is associated with a better prognosis, whereas the presence of other TILs is associated with a poor prognosis [25]. For example, in colorectal cancer, the infiltration of T_H_1 cells and an IFN-*γ* signature is associated with a good prognosis, whereas T_H_17 lymphocytes are associated with a poor prognosis [26]. T_H_17 cells secrete large amounts of interleukin-17 (IL-17), and this cytokine is also the most important cytokine in chronic inflammation, linking CRC to inflammation. As the TME depends on the inflammatory processes that are influenced by the molecular phenotype and the stage of the CRC, the TME is very dynamic and can change dramatically from anti-tumor features to pro-tumor features and vice versa [27]. Tumor-promoting or tumor-destructive features are mediated by inflammatory soluble factors, mainly cytokines and chemokines [28,29]. Long-term inflammation can lead to cancer, as seen in colitis-associated cancer (CAC) [29]. Although CAC is preceded by the long-term inflammation of the bowel (inflammatory bowel disease, IBD), this colorectal cancer shows a similar number of somatic mutations as sporadic CRC [30]. This contradicts the assumption that chronic inflammation increases the number of CRC-inducing mutations. Although the quantity of mutations are more or less the same in colitis-associated and sporadic colon cancers, CAC and sporadic CRC harbor different mutations in important tumor-associated genes such as suppressor of cytokine signaling 1 (SOCS1), p53 or genes of the Wnt signaling pathway [31]. However, not only qualitative differences in the mutational landscape of CAC and sporadic CRC exist, CAC show cytokine-driven epigenetic differences in genes of the Wnt pathway leading to the hyperactivation of Wnt signaling [30]. Therefore, it is conceivable that the effects of chronic inflammation together with qualitative mutations in tumor-suppressor genes or oncogenes lead to colitis-associated cancer and not the quantity of the mutations.

We want to mention that a recent study analyzing single cell transcriptomes from 63 patients with a focus on epithelial cells and fibrosis identified two epithelial tumor states and refined the CMS classification. The two distinct epithelial subtypes corresponding to the CMS classification were intrinsic (i) CMS2 and iCMS3. Most CMS2 and CMS3 tumors contained iCMS2 and iCMS3 epithelial cells. MSI-H and CMS1 cancers were classified iCMS3. MSS tumors with iCMS3 epithelium (iCMS3_MSS) had features that were more similar to MSI-H than to iCMS2_MSS cancers. The fibrotic CMS4 group comprised two epithelial subtypes. Five functional subtypes of CRC were defined, based on epithelial type, microsatellite status and presence of fibrosis [32]. This new refined classification lays the focus on epithelial cells and fibrosis. However, cells of the TME and not epithelial cells are the main producers of cytokines. Thus, the TME influence is better described by the “established” CMS classification outlined in Figure 2.

Here, we discuss how cytokines can influence all cells of the colorectal tumor eliciting an inflammatory milieu that can either promote cancer growth or diminish CRC growth. We show how the kind of inflammation with different TME compositions and different cytokines influences tumor progression, invasion and therapeutic opportunities and discuss new treatment strategies. 

### 1.3. Interleukins, Interferons and other Cytokines in CRC

Interleukins (ILs) are small proteins that can be divided in several families with more than 40 subfamily members. In the tumor, inflammatory interleukins from the same family can either activate immune responses or suppress immune responses against the tumor. In CRC most cytokines mediate a more pro-tumorigenic effect. For example, IL-6, a pro-inflammatory cytokine secreted by, e.g., monocytes and macrophages, inhibits apoptosis and the release of reactive oxygen species (ROS). Under homeostatic conditions, this cytokine, together with IL-10, IL-11 and IL-23, functions as an alarmin to resolve inflammation [33]. Other alarm cytokines such as IL-1*α* and IL-1*β* initiate and intensify local inflammation in response to DAMPs and PAMPs by pathogen-recognition receptors [34]. IL-1*α* and IL-1*β* are significantly increased in CRC while another cytokine of the Il-1 family, IL-18, is decreased in CRC patients, suggesting a prominent anti-tumorigenic role in colorectal cancer [34]. The IL-2 family consists of several cytokines with tumor-promoting and tumor-suppressive effects. IL-2, IL-9 and IL-15 show tumor-suppressive effects in CRC, while IL-4 and IL-7 exert pro-tumorigenic effects [35]. The IL-10 family consists of six cytokines with IL-10, IL-19, IL-20, IL-22 and IL-26 described as tumor promoting. Only one cytokine of this family, IL-24, was described as tumor preventing [33]. In contrast, IL-12 induces the activation of T_H_1 cells in the TME of CRC and the subsequent activation of NK cells [36]. The inflammatory cytokine TNF plays a major role in CRC and is one of the earliest and most important pro-inflammatory cytokines that activates other pro-inflammatory cytokines via the NF-*κ*B signaling pathway. TNF, together with IL-6, IL-8, and IL-17A, increases the Wnt/*β*-catenin signaling and promotes intestinal tumorigenesis [37]. The transforming growth factor beta (TGF-*β*) is a pro-inflammatory cytokine that plays a substantial role in tumor initiation and progression in CRC [36]. Tumors of the CMS4 subtype are characterized by high TGF-*β* levels expressed mainly by fibroblasts in the CMS4 tumor microenvironment [38].

Interferons (IFNs) are divided into three subtypes: (1) type I IFNs with IFN-*α*, IFN-*β*, IFN-*ε*, IFN-*κ* and IFN-*ω*, (2) type II with IFN-*γ* and (3) type III with IFN-*λ*. All type I IFNs signal through the IFN-*α*/*β* receptor (IFNAR) and are induced by DAMPs and PAMPs [39]. Type I IFNs are crucial for antigen-presenting cells to prime efficient T cell responses as they promote the differentiation of T_H_1 cells and enhance the killing capacity of NK cells (for a detailed review see [39]). IFN-*γ* is the only member of the type II subtype. Due to epigenetic, transcriptional, post-transcriptional and post-translational regulation, only immune cells and effector T cells produce IFN-*γ* [40]. In the TME, the classical producers of IFN-*γ* are T_H_1 cells, CTLs and NK cells. Although only a few cells in the TME produce IFN-*γ*, all nucleated cells constitutively express the IFN-*γ* receptor. This pleiotropic expression of the receptor leads to numerous effects depending on the composition of the TME. However, the infiltration of cells that produce high levels of IFN-*γ* in the TME of CRC is associated with longer disease-free survival and overall survival [41]. Colorectal tumors that fall into the immune infiltrated and activated CMS1 phenotype express high levels of IFN-*γ* [24], correlating to a good prognosis in CRC, although the infiltrating T cells show high expression of PD-1 and CTLA-4 [38]. In contrast to CMS1 tumors, CMS4 tumors lack the expression of IFN-*γ* and the infiltration of T and B cells. The last IFN, IFN-*λ*, has not been described in the context of CRC yet. 

### 1.4. Chemokines in CRC

The group of chemokines consists of 50 chemoattractant chemokines divided into different subfamilies. Chemokines, therefore, constitute the largest subgroup of cytokines. Chemokines recruit tumor-infiltrating cells into the tumor and are, thus, key mediators of inflammation in cancers. Owing to their multidirectional and pleiotropic regulatory networks, there is no chemokine that gives the same prognosis in all types of cancers. For example, the increased expression of the chemokine CCL-2 leads to a bad prognosis in CRC, whereas the overexpression of the same chemokine leads to a good prognosis in breast cancer [42]. Chemokine receptors are pleiotropic, which means that a special chemokine receptor can bind multiple ligands. Conversely, several chemokines can bind to more than one receptor. For example, the chemokine receptor CXCR2 can bind the chemokines CXCL-1, CXCL-2, CXCL-3, CXCL-5, CXCL-7 and in humans also the chemokines CXCL-6 and CXCL-8 [43]. The chemokine CCL-2 can bind to CCR2 and CCR5. In CRC, the infiltration of CD8^+^ CTLs is associated with a longer disease-free survival [44]. CD8^+^ CTLs express the chemokine receptor CXCR3 and, therefore, are recruited by CXCL-9, CXCL-10 and CXCL-11. Transcriptional studies have linked the chemokines CXCL-1, CXCL-9, CXCL-10, CCL-2, CCL-5 and CCL-11 with T cell infiltration in CRCs [45]. CMS1 tumors show a high expression level of the chemokines CXCL-9, CXCL-10 and CXCL-16, all chemokines that are associated with T cell trafficking and activation [24]. The chemokines CCL-2 and CCL-5 attract macrophages into the tumor and lead to tumor progression and metastasis [46]. The mesenchymal consensus molecular phenotype 4 (CMS4) tumors are characterized by high expression of CCL-2 and CXCL-12. Consequently, CMS4 tumors show a high density of macrophages, MDSCs and fibroblasts [38]. In this regard, the secretion of the chemokine CCL-2 can not only attract macrophages but can also polarize them into an pro-tumoral M2 phenotype [47]. As far as the most abundant cell type of blood immune cells is concerned, the chemokines CXCL-1, CXCL-2, CXCL-3, CXCL-5, CXCL-7 and CXCL-8 recruit neutrophils into colorectal tumors [46]. Coming back to T cells, Tregs express high amounts of CCR4 and infiltrate the TME in response to CCL-22, secreted by TAMs. On the other hand, myeloid derived suppressor cells are recruited by CCL-2, CXCL-5, CXCL-8 and CXCL-12 into the TME, building a tumor-promoting and immune-suppressive microenvironment. The chemokine CXCL-13 attracts B cells and T follicular helper (T_FH_) cells in secondary lymphoid structures and in tertiary lymphoid structures. CMS1 tumors show a high expression level of CXCL-13 and, therefore, high densities of B cells and T_FH_ cells. Both cell types are associated with a good prognosis in CRC [38]. In contrast to CMS1, the CMS4 phenotype expresses very low levels of CXCL-13 and, therefore, lacks secondary and tertiary lymphoid structures. CRC patients with a mutation in the CXCL-13 gene have a shorter survival time [38]. However, the tumor-promoting or tumor-preventing effect of a specific chemokine is not entirely clear, as chemokines can also activate inhibitory pathways that are crucial in preventing tumor growth [48]. The chemokine receptor CXCR4 is most often expressed in more than 23 human cancers, including CRC. CXCL-12 is the ligand for CXCR4. The CXCL-12/CXCR4 axis correlates with tumor growth, invasion, angiogenesis and metastasis in CRC, making this chemokine axis to one of the most promising targets for an inhibitory therapy [49].

## 2. Cytokine-Induced Inflammation

### 2.1. Cytokines in Gut Homeostasis and in Chronic Inflammation

Due to a variety of different functions such as nutrient absorbance, digestion, waste excretion, barrier regulation and interactions with the microbiome, most of the intestinal tract consists of only two cell types: intestinal epithelial cells (IECs) and immune cells. IECs have a short life span of only 4–5 days and are constantly renewed by stem cells from the bottom of the crypt. On the tip of the villus, cytokines such as tumor necrosis factor (TNF) induce apoptosis and the cell sheds into the lumen [50,51]. The major cell type of IECs are absorptive enterocytes, followed by anti-microbial peptide-producing Paneth cells (in the small intestine only), mucus-producing goblet cells and other cell types. Immune cells of the intestine are mostly harbored in gut-associated lymphoid tissues but are also present in the lamina propria. Whereas immune cells in the lamina propria are cells of the innate and adaptive immune system, immune cells in the epithelium are only T cells called intraepithelial lymphocytes (IELs). The intestinal epithelium faces a great challenge; on the one hand, it must permit nutrient absorbance, and on the other hand, the epithelium must maintain an impermeable barrier to microorganisms and antigens. Cells communicate via cytokines and chemokines and therefore both factors are critical for mucosal homeostasis. To maintain homeostasis in the gut, cytokines from the IL-1 group, such as IL-1*α*, IL-1*β* and IL-18, play an important role. IL-1*α* is constantly expressed in an inactive form by IECs, whereas IL-1*β* expressed by mononuclear cells in the lamina propria is inducible. After damage of the mucosal barrier, IL-1*α* is activated and acts as an alarmin [52]. Several studies showed that IL-1*α*-deficient mice exhibited reduced severity of intestinal inflammation, suggesting a pathogenic role in intestinal inflammation [53,54,55]. Unlike IL-1*α*, IL-1*β* plays a more dichotomous role as it either promotes or disrupts the barrier integrity, depending on the inflammatory mouse model [55,56,57]. IL-1*β* expression leads to an accumulation of pathogenic IL-17A-secreting immune cells, mediating chronic inflammation [58]. Another cytokine of the IL-1 family is IL-18. Similar to IL-1*α*, IL-18 is constitutively expressed as an inactive precursor molecule by mononuclear cells, endothelial cells, keratinocytes and epithelial cells [57]. In addition, similar to IL-1*β*, IL-18 was described to both enhance and disrupt the epithelial barrier integrity [59]. Nonetheless, IL-18 expression seems to be important for epithelial integrity during homeostasis as overexpression leads to experimental colitis. Due to the short life span of IECs, several cytokines stimulate intestinal epithelial proliferation. Although the pro-inflammatory cytokines TNF, IL-6 and IL-17 contribute to the pathology of tumor-elicited inflammation, these cytokines are crucial for replacing epithelial cells by stimulating the proliferation [60,61,62]. Despite TNF, IL-6 and IL-17, several other cytokines promote the proliferation of epithelial cells. IL-22 induces the proliferation of stem cells [63] and is necessary for stem cell maintenance [64]. IL-36 is an important cytokine in wound healing by increasing the proliferation of epithelial cells adjacent to the wound [65]. The cytokines IL-13, IL-4 and IL-33 induce the differentiation of tuft and goblet cells from progenitor cells [66,67].

Inflammation is a physiological response to injuries and begins with the secretion of biomolecules from damaged tissues. After the infiltration of white blood cells, the wound heals and the signaling cascade ends. In chronic inflammation, the signaling cascade proceeds although there is no injury present any longer [68]. The correlation between cancer and chronic inflammation was first observed by Rudolf Virchow more than 150 years ago [69]. Chronic inflammation leads to epithelial–mesenchymal transition, dedifferentiation, upregulated reactive oxygen species (ROS) and cytokine levels and epigenetic changes in tumor and in stromal cells [70]. 

Chronic inflammation seems to boost tumor growth and enhance early tumorigenesis in organs that have contact with microbial products or directly with microbiota [71]. As an example, *Helicobacter pylori* infection is known to induce chronic inflammation that often leads to gastric cancer [72,73]. In colorectal cancer, the bacterial strains *Chlostridioides difficile* and its toxin TcdB promote colonic carcinogenesis in a germ-free mouse model of colorectal cancer [74]. The tumor promoting ability of some bacteria is mediated by inflammatory cells of the tumor microenvironment and the cytokines these cells produce [75]. Long-term reduction or prevention of chronic inflammation by aspirin or other nonsteroidal anti-inflammatory drugs reduces CRC incidence and mortality [76], highlighting the importance of inflammatory cytokines.

Inflammation starts with an insult caused by environmental factors such as obesity, smoking, inhaled pollutants or infections with pathogenic bacteria or aberrant immune reactions. In the colon, the epithelial barrier becomes leaky, and bacteria can invade into deeper layers of the mucosa. This activates neutrophils to produce IL-1*α* and IL-18 [77]. At the same time, the inflammasome is activated and damage-associated molecular patterns (DAMPs) and pathogen-associated molecular patterns (PAMPs) are recognized via Toll-like receptors on monocytes, macrophages and lamina-propria DCs and enhance the inflammation of the mucosa by the production of large amounts of the pro-inflammatory cytokines IL-1*β*, IL-6, IL-18 and TNF [78]. These pro-inflammatory cytokines increase the permeability of the vessels, leading to the accumulation of monocytes and lymphocytes that produce several pro-inflammatory cytokines, such as IFN-*γ*, TNF, IL-17A, but also immunoregulatory cytokines, such as IL-10, IL-12 and IL-23, that suppress adaptive immunity and prevent over-stimulation of the immune system. Meanwhile, epithelial cells migrate into the wound and begin to close it. The migration and re-differentiation of epithelial cells is dependent on the cytokine TGF-*β* [79]. In addition to interleukins, several chemokines, small secreted proteins that mediate immune cell trafficking, are produced in the inflammatory milieu. For example, monocytes are recruited by the chemokines CC-chemokine ligand 2 (CCL-2) or CCL-5, T cells and NK cells by CCL-20, CXCL-9, CXCL-10 and CXCL-11 and immature DCs by the chemokine CCL-20 [43].

Inflammation and resolution of the inflammation are very complex processes involving several immune cell types, epithelial cells and numerous cytokines. If the inflammation cannot be resolved and the mucosal barrier is unrepairable, cytokines that help tumor cells to proliferate, such as IL-36, TNF, IL-6 or IL-17, or cytokines that reduce the activity of tumor-eliminating immune cells, such as IFN-*γ* or TNF, are already at the site of tumor development. Although inflammation alone is not capable of beginning the malignant transformation of the epithelial cells, the pro-inflammatory cytokines induce epigenetic changes, and deoxyribonucleic acid (DNA) breaks via upregulated ROS levels, dedifferentiation of epithelial cells, infiltration of myeloid cells and migration via EMT [70]. Together, these events lead to a pro-tumorigenic microenvironment.

### 2.2. Cytokines in Tumor-Elicited Inflammation

Cytokines are classified into pro-inflammatory cytokines and chemokines, anti-inflammatory cytokines and chemokines and cytokines and chemokines with a mixed phenotype, depending on the target cell. Table 1 summarizes the cytokines and their receptors.

CRCs arise from precursor polyps, mostly from the two most common polyps, pre-cancerous conventional adenomas and sessile serrated polyps [80]. One hallmark of tumorigenesis is the accumulation of mutations or epigenetic alterations. These mutations and alterations lead to the activation of oncogenes or the inactivation of tumor suppressor genes. However, this is not enough for malignant cell transformation, as some studies have shown that phenotypically normal cells can carry several somatic mutations without developing into cancer cells [81,82,83,84]. For the progression of normal cells into malignant cells with uncontrolled infinitive proliferation capacity, cells of the microenvironment have to support and promote tumorigenesis by pro-inflammatory cytokines. As mentioned in the previous paragraph, inflammatory cytokines support successful tumor development, transformation and progression of mutated cells. In addition, as also mentioned in the previous paragraph, most pro-inflammatory cytokines are necessary for gut homeostasis and wound healing. Examples for these dichotomous functions are IL-1*α* and IL-1*β*. As alarmins that sense DAMPs and PAMPs, IL-1*α* and IL-*β* activate a cascade of cytokines. This cascade plays a major role in the activation and orchestration of innate and adaptive immunity. Many of the intrinsic (oncogene-driven) and the extrinsic (TME-driven) tumor-promoting effects are mediated by IL-1 [85]. IL-1*β* mediates cell proliferation, differentiation and apoptosis; stimulates the expression of TNF, IL-6, IL-8, and IL-17A; and increases Wnt/*β*-catenin signaling, promoting intestinal tumorigenesis [37]. After a localized inflammatory process with the secretion of IL-1 cytokines from, e.g., macrophages and neutrophils, CRC tumor cells can create a positive feedback loop, promoting the expression of more pro-inflammatory cytokines that stimulate cancer-cell proliferation and drug resistance [36]. The regulation of cytokines is quite complex as cytokines are not only regulated by transcriptional and post-transcriptional mechanisms but also by the availability of cytokine receptors, which are also regulated transcriptionally and post-transcriptionally.

**Table 1:** Cytokines in colorectal cancer.

**Cytokines that Promote CRC**				
Cytokine Family	Cytokine	Receptor	Functional effect in CRC	Reference
IL-1 Family	IL-1*α*	Interleukin 1 receptor type 1 (IL-1R1)-IL-1R3	Promotes inflammatory carcinogenesis	[86]
	IL-1*β*	IL-1R1-IL-1R3IL-1R2-IL-1R3	Promotes the proliferation of colon cancer cells, promotes inflammation-induced carcinogenesis	[86,87]
IL-2 (common *γ*-chain) Family	IL-4	IL-4R	Promotes Th2-type inflammation and Th9 polarization	[88,89]
IL-6 Family	IL-6	IL-6R*α*-gp130	Promotes carcinogenesis via upregulation of proliferation, mitosis, migration and angiogenesis	[90,91,92]
	IL-11	IL-11R*α*-gp130	Promotes inflammation-induced carcinogenesis, facilitates the proliferation of colon cancer cells	[93,94,95]
	IL-31	IL-31R*α*-OSMRb	Promotes Th2 cell polarization, evidently tumorigenic	[96]
IL-8 Family	IL-8	CXCR1, CXCR2	Promotes colon cancer cell proliferation, attracts neutrophils, mediates a suppressive environment and chemoresistance	[97,98,99,100]
IL-10 Family	IL-19	IL20R*α*-IL20R*β*	Evidently tumorigenic	[101]
	IL-20	IL-20R*α*-IL-20R*β*	Promotes carcinoma outgrowth, induces PD-1	[102]
	IL-22	IL-22R*α*-IL-10R*β*	Promotes progression of carcinomas, stemness and proliferation	[103,104,105]
	IL-26	IL-20R*α*-IL-10R*β*	Promotes T_H_17 polarization, only expressed in humans, not in mice	[102]
IL-12 Family	IL-23	IL-23R-IL-12R*β*	Promotes pro-inflammatory cytokine secretion	[106,107]
	IL-35	IL-12R*β*-gp130 IL-12R*β*-IL-12R*β*	Promotes Treg-mediated suppression of T cell responses and exhaustion of T cells	[108]
IL-17 Family	IL-17A	IL-17RA-IL-17RC	Promotes cell cycle progression of colon cancer cells and immunosuppression	[109,110,111]
Other cytokines				
	IL-13	IL-13R*α*-IL-4R*α*	Promotes Th2 cell polarization	[112]
	IL-16	CD4	Evidently pro-tumoral	[113]
	IL-34	CSF1R	Promotes cancer progression and immune suppression and therapeutic resistance	[114,115,116]
	TNF	TNFR1, TNFR2	promotes inflammation	[117,118,119]
Chemokines	CXCL-1	CXCR1, CXCR2	Recruitment of tumor-associated macrophages (TAM) and TANs	[120,121]
CXC chemokines	CXCL-2	CXCR2	Recruitment of TAMs and TANs	[122]
	CXCL-8	CXCR1, CXCR2	Recruitment of TANs, TAMs and cancer cells to the tumor site; promotion of angiogenesis and tumor stemness	[123,124,125]
	CXCL-12	CXCR4, CXCR7	Recruitment of TAMs at the invasive front, upregulation of IL-10	[126,127]
	CXCL-16	CXCR6	Induces the polarization of macrophages toward a pro-tumoral M2 phenotype	[128]
CC chemokines	CCL-2	CCR2	Recruitment of TAMs at the invasive front, recruitment of myeloid-derived suppressor cells (MDSC) into the tumor	[129,130]
	CCL-3	CCR1, CCR4	Recruitment of MDSC into the tumor, promotes proliferation of colon cancer cells	[131]
	CCL-4	CCR1, CCR3	Recruitment of MDSC and TAMs into the tumor	[132]
	CCL-11	CCR8	Promotes tumor angiogenesis, inhibits apoptosis of endothelial cells	[133]
	CCL-16	CCR1, CCR2, CCR3, CCR5, CCR8	Promotes tumor angiogenesis, inhibits apoptosis of endothelial cells	[134]
	CCL-17	CCR4	Recruitment of Tregs and Th2 lymphocytes	[135]
	CCL-20	CCR6	Recruitment of Tregs and Th2 lymphocytes	[136,137]
**Cytokines with a more CRC-suppressive phenotype**				
Cytokine Family	Cytokine	Receptor	Functional effect in CRC	Reference
IL-1 Family	IL-18	IL-5R5-IL-1R7	Activates lymphocytes to produce IFN-g, restricts Th_H_17 differentiation	[138,139]
	IL-36	IL-1R6-IL1R3	Conservation of tertiary lymphoid structures	[34,140,141]
	IL-37	IL-1R8-IL-1R5	Inhibits β-catenin	[141,142]
IL-2 (common *γ*-chain) Family	IL-2	IL-2R	T and NK cell activation factor	[34,143]
	IL-7	IL-7R*α*	Promotes the proliferation of T cells and NK cells	[144,145]
	IL-9	IL-9R*a*	Promotes the proliferation of CD8^+^ T cells	[146,147]
	IL-15	IL-15-IL-15R*α*	Promotes the proliferation and activation of NK cells and CD8^+^ T cells	[148,149,150]
	IL-21	IL-21R	Promotes the proliferation and activation of NK cells and CD8^+^ T cells	[151,152,153]
IL-10 Family	IL-24	IL-20R*α*-IL-20R*β*, IL-22R*α*-IL-20R*β*	Induces apoptosis and autophagy of cancer cells	[154]
IL-12 Family	IL-12	IL-12R*β*	Promotes T cell survival and proliferation and the proliferation of NK cells, enhances cytotoxic function	[155,156]
IL-17 Family	IL-17F	IL-17RA-IL-17RC	Inhibition of tumor angiogenesis, enhancing immune cell recruitment	[34,157]
Other cytokines				
Interferon Family	IFN-γ	IFNGR	Mediates anti-proliferative, anti-angiogenic and pro-apoptotic effects	[44,158,159]
	Type I interferons	Interferon alpha and beta receptor subunit 1 (IFNAR1)-IFNRA2	Recruitment of CD8^+^ lymphocytes	[160]
TGF-β	TGF-β	TGFBR1-TGFBR2	Inhibits cancer cell proliferation, regulates immune cell differentiation	[161,162]
Chemokines				
CXC chemokines	CXCL-9	CXCR3	Promotes the infiltration of NK cells, CD8^+^ and CD4^+^ T cells into the tumor, suppression of angiogenesis	[163,164]
	CXCL-10	CXCR3	Promotes the infiltration of NK cells, CD8^+^ and CD4^+^ T cells into the tumor, suppression of angiogenesis	[160,163,165]
	CXCL-11	CXCR3	Promotes the infiltration of NK cells, CD8^+^ and CD4^+^ T cells into the tumor, suppression of angiogenesis	[166]
**Cytokines with a mixed phenotype**				
Cytokine Family	Cytokine	Receptor	Functional effect in CRC	Reference
IL-1 Family	IL-33	IL-1R4	Alters TME, promotes angiogenesis, enhances colon cancer stemness but induces anti-tumoral IFN-γ responses	[167,168,169,170]
IL-2 (common *γ*-chain) Family	IL-9	IL-9R	Inhibits tumor growth by enhancing immune responses and promotes tumor growth by suppressing immune responses	[112,171]
IL-10 Family	IL-10	IL-10R*α*-IL-10R*β*	Promotes cytotoxicity but inhibits anti-tumor responses	[172]
Chemokines				
CXC chemokines	CXCL-4		Suppresses the activity of CD8^+^ T cells and inhibits tumor angiogenesis and endothelial cell proliferation	[173]
	CCL-5	CCR1, CCR3, CCR4, CCR5	Recruitment of MDSC and fibroblasts into the tumor but also CD8^+^ T cells	[160,174,175]

#### 2.2.1. Cytokines in the Early Stages of CRC

**Figure 3:**
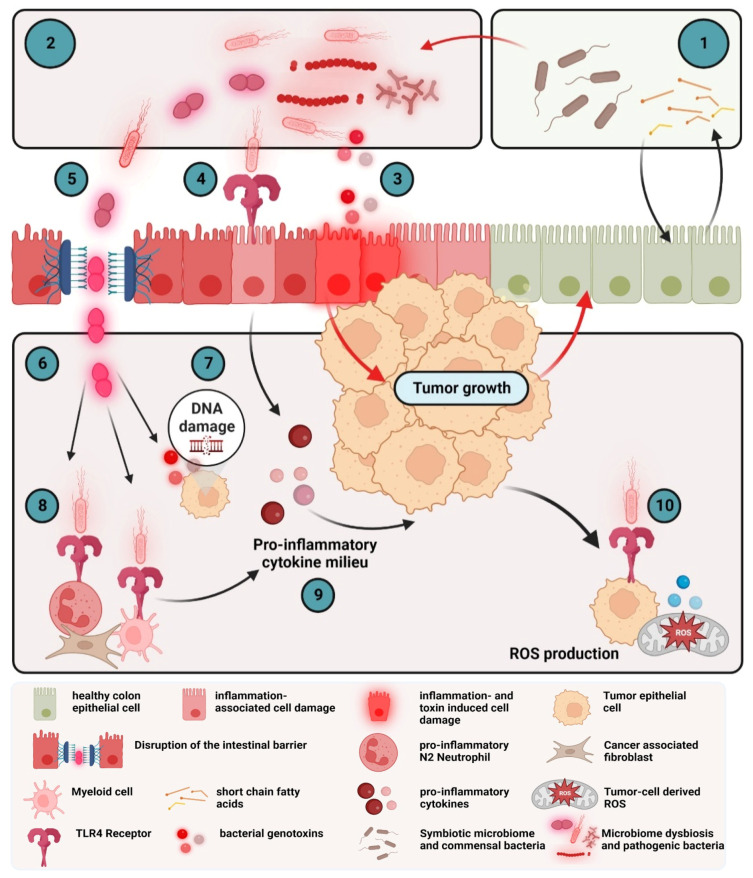
Microbiota-induced pro-inflammatory cytokine milieu in colorectal cancer. The microbiome of a healthy individual is characterized by a high species diversity (1). The microbiome in colorectal cancer is characterized by dysbiosis with an overrepresentation of pathogenic bacteria and their metabolites (2). Bacterial metabolites can induce hyperactivation of the WNT-*β*-catenin pathway and secretion of TNF and IL-17 in epithelial cells (3). Bacteria interact with gut epithelial cells via pattern recognition receptors such as TLRs. Activation of TLRs by PAMPs leads to expression of pro-inflammatory cytokines such as IL-1*β* (4). Some pathogenic bacteria increase the intestinal permeability (5), leading to the translocation of bacteria (6) Bacterial metabolites of the invading bacteria induce DNA damage and, thus, direct mutagenic effects (7). Myeloid cells sense bacterial products via TLR and produce several pro-inflammatory cytokines, such as IL-6, TNF or IL-17, that further promote tumor growth (8 and 9). Binding to TLR4 on tumor cells leads to ROS production, further promoting the circulus vitiosus between dysbiosis, invasion, inflammation and tumor growth (10).

Disruption of the epithelial barrier by bacterial infection, microbial metabolites, obesity or epithelial damage results in the production and release of several pro-inflammatory cytokines. However, even the alteration in gut microbiota (Figure 3) can promote tumorigenesis as the microbiome and the intestinal epithelial cells interact in a complex network to maintain homeostasis [176]. The gut microbiome of a healthy individual is characterized by a high species diversity of, e.g., *Bacteroidetes*, *Firmicutes* and *Actinobacteria* [177,178]. Colorectal cancer is associated with a dysbiosis of this diversity with an overrepresentation of specific bacteria such as the anaerobic bacterium *Fusobacterium nucleatum* [179], *Streptococcus gallolyticus*, *Bacteroides fragilis*, *Escherichia coli* or *Enterococcus faecalis* [180]. Gut bacteria can influence tumor growth or inhibition of CRC proliferation through direct cell interaction and through microbial-derived metabolites [181]. The most well studied metabolites are the short-chain fatty acids (SCFAs). SCFA metabolism by epithelial cells maintains the hypoxic environment of the colon that most bacteria require. In the absence of SCFA, epithelial cells switch to anaerobic respiration, release oxygen and nitrates into the colon lumen and fuel the expansion of pathogens such as *Escherichia coli* [182]. *Escherichia coli* is a Gram-negative bacterium with a membrane that consists of lipopolysaccharides (LPS). LPS bind to the Toll-like receptor (TLR) 4 on many immune and some epithelial cells and induce the secretion of pro-inflammatory cytokines. TLRs are a family of pattern-recognition receptors mostly expressed on the surface of cells of the innate immunity such as granulocytes. SCFAs prevent tumorigenesis by inhibiting LPS-induced expression of pro-inflammatory cytokines and induce colon adenoma and carcinoma cell apoptosis [183]. SCFAs decrease the two important CRC signaling pathways nuclear factor kappa-B (NF-*κ*B) and Wnt, facilitating anti-tumorigenic effects, and reduce pro-inflammatory cytokines such as TNF, IL-6 or CCL-3 [181]. Some pathogenic bacteria are able to invade the epithelium directly by increasing the intestinal permeability. This is achieved by binding to the tight junction proteins such as E-cadherin, occludin or claudin1. *Fusobacterium nucleatum* can bind to E-cadherin and invade the epithelial cell, thus, promoting colon cancer [184]. 

There are some more microbial modulators of tumor inflammation such as microbial toxins that activate immune responses and inflammation. For example, the *Bacteroides fragilis* toxin (BFT) and the colibactin from *Eschericchia coli* induce DNA damage in epithelial cells (referred as genotoxins) and, subsequently, a strong T_H_17 response [185,186]. Conversely, bacterial adhesins such as FadA from *Fusobacterium nucleatum* or AFA-1 from *Eschericchia coli* can bind E-Cadherins on the surface of epithelial cells and activate *β*-catenin signaling and, subsequently, the release of IL-6 and TNF [187].

Disrupted intestinal barriers at the site of a small colorectal tumor induce the activation of innate immune cells and the increased expression of pro-inflammatory cytokines. One of the major cytokines that fuels CRC progression is TNF. TNF is one of the earliest and most important pro-inflammatory cytokines that activates other pro-inflammatory cytokines via the NF-*κ*B signaling pathway. TNF has also contradictory effects in cancer progression due to its two receptors. TNFR1 has a cytoplasmic death domain so that binding of TNF to the TNFR1 can lead to apoptosis [188]. In CRC, the apoptosis-inducing properties of TNF are less pronounced compared with the pro-inflammatory properties that induce the production of several inflammatory cytokines, enhanced oncogene activation, tumor cell invasion and migration and creation of a tumor-supportive TME [172]. 

Concomitant with or shortly after the secretion of TNF, the cytokines IL-1 and IL-6 are produced. As described in the former paragraphs, the alarmins IL-1*α* and IL-1*β* mediate cell proliferation, differentiation and apoptosis, stimulate the expression of TNF, IL-6, IL-8, and IL-17A, and increase the Wnt/*β*-catenin signaling [37].

The cytokine IL-6 is involved not only in cancer inflammation but also in hematopoiesis, bone metabolism and embryonic development [92]. The IL-6R is composed of the binding receptor chain IL-6Ra and the signal transducer chain glycoprotein 130 (gp130). As IL-6 is a target of the transcription factor NF-*κ*B, the activation of NF-*κ*B by, e.g., TNF, simultaneously with the activation of signal transducer and activator of transcription 3 (STAT3) in non-immune cells such as epithelial cancer cells, triggers a positive feedback loop by the IL-6-signal transducer and activator of transcription 3 (STAT3) axis [68]. The resulting excessive activity of IL-6 leads to the overexpression of the proto-oncogene c-Myc, multiple pro-inflammatory cytokines and, consequently, tumor growth, tumor progression and drug resistance. At the same time, IL-6 suppresses anti-tumorigenic immune responses [189]. In the early stages of CRC, chemokines shape the composition of the TME. The chemokine IL-8 attracts neutrophils and can be induced by TNF. T_H_17 cells express elevated levels of the C-X-C chemokine receptor type 4 (CXCR4), the receptor of CXCL-12 and CC chemokine receptor 6 (CCR6), the receptor of CCL-20. The chemokines that attract monocytes are CCL-2 and CXCL-5 [43]. Thus, at the early phase of colorectal cancer, cytokines change the balance as the TME develops to a more tumor promoting or tumor suppressing immune microenvironment.

#### 2.2.2. Cytokines in the Late Stages of CRC

**Figure 4:**
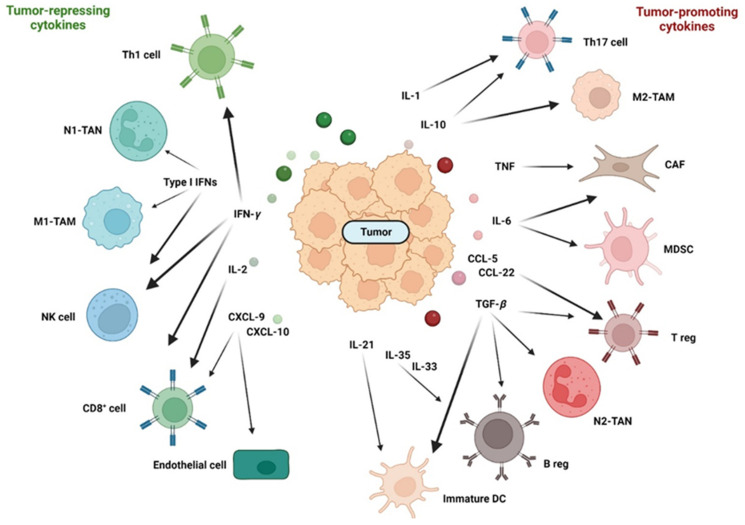
The relationship between tumor repressing and tumor-promoting cytokines in the TME. Stress, bacteria and their components that cross disrupted barriers or death of tumor cells induce inflammation with increased expression of several cytokines and chemokines. Depending on the composition of the cytokine/chemokine mixture, a tumor microenvironment that is more immunosuppressive and tolerogenic (**right**) or more tumor suppressive (**left**) is induced.

As the tumor progresses, more immune cells enter the tumor, attracted by chemokines. TNF and IL-1 synthesized by leukocytes renders endothelial cells into pro-inflammatory endothelial cells that secrete a large amount of chemokines, such as IL-8 and CXCL-2, to further enhance the infiltration of neutrophils. As neutrophils are the first responders against extracellular pathogens, neutrophils were originally considered to be defensive against colorectal cancer [190]. However, neutrophils in CRC can promote the growth of cancer cells by regulating the innate and adaptive immune system and inducing angiogenesis [191]. In the TME, neutrophils can differentiate into tumor-associated neutrophils (TANs) with a N1 or N2 phenotype (see Figure 4 for an overview of cells and cytokines of the TME). N1 TANs have a more anti-tumorigenic phenotype, whereas N2 TANs have a more tumor-promoting phenotype. TGF-*β* polarizes toward a N2 phenotype that produces several chemokines such as CCL-2, CCL-5 and CXCL-4 [191]. IFN-*β* polarizes toward an N1 phenotype [192].

Fibroblasts play a key role in wound healing as they are the major producers of the extracellular matrix. Although cancer-associated fibroblasts (CAFs) are a central component of the TME of nearly every solid tumor, they comprise a heterogeneous population of cells with no precise fibroblast-specific markers. Cells that are negative for epithelial, endothelial and leukocyte markers with an elongated morphology and lacking the mutations of cancer cells are considered to be CAFs [193]. In the TME, various inflammatory cytokines such as IL-1, TNF or IL-6 can activate fibroblasts to become CAFs. CAFs secrete numerous cytokines and chemokines, such as TGF-*β*, IL-6, IL-1β, IL-4, and CXCL-12 (SDF-1), which influence CD8^+^ T cells, Tregs and macrophages to act as immuno-suppressive or immuno-promoting cells. In CRC, CAFs have more immuno-suppressive effects and consequently promote cancer cell proliferation and therapy resistance [194].

Tumor-associated macrophages (TAMs) also play a major role in the TME of CRC. Macrophages eliminate invading microbes by phagocytosis, in homeostasis resident macrophages in the colon secrete high amounts of IL-10 and are, therefore, anti-inflammatory [195]. Under inflammatory conditions, inflammatory CX3CR1-positive macrophages enter the colon and secrete the pro-inflammatory cytokines TNF and IL-6 [196]. Similar to neutrophils, macrophages can differentiate into two different phenotypes. The classically activated M1 phenotype has the capability to promote T helper 1 (T_H_1) responses and kill tumor cells. M1 differentiation is induced by bacterial components, IFN-*γ* and TNF. In contrast, alternatively activated M2 macrophages display tumor-promoting activity and immuno-suppressive functions. M2 differentiation is induced by IL-6, IL-10 or IL-13. M2 macrophages are typically divided into four subtypes, which are differently stimulated: the M2a, induced by IL-4 and IL-23; the M2b subtype, stimulated by IL-1; the M2c subtype, induced by IL-10 and the M2d macrophage, induced by IL-6. Through the expression of the two immuno-suppressive cytokines IL-10 and TGF-*β*, M2 macrophages directly induce an immunosuppressive microenvironment [197,198]. Through the secretion of IL-6, M2 macrophages mediate the expression of IL-10 by colorectal cancer cells and indirectly induce a microenvironment that suppresses T cell activity [199]. The partition of macrophages in the two subtypes M1 and M2 is a simplified view; in reality, TAMs exist in various states between the two phenotypes.

Macrophages and neutrophils belong to the group of tumor-infiltrating myeloid cells (TIMs), a heterogeneous population of cells characterized by diversity and plasticity. One member of this myeloid cell group is myeloid-derived suppressor cells (MDSCs). MDSCs are able to most effectively suppress T cell activities by secreting high amounts of IL-10 and TGF-*β* [200]. MDSCs are very plastic as they can differentiate into TAMs or tumor-associated DCs, depending on the signals in the TME [201].

T cells are the most abundant in the TME and can be divided into CD8^+^ cytotoxic T lymphocytes (CTLs), CD4^+^ T helper cells, regulatory T cells and NKT cells.

CD8^+^ CTLs are effector cells of the adaptive immune system and play a pivotal role in anti-tumorigenic immune responses by direct killing of malignant cells. CTLs in the TME of CRC produce high amounts of IL-2, IL-12 and IFN-*γ* that activate the killing efficiency of NK cells and CTLs [202] and enhance the expression of CXCL-9, CXCL-10 and CXCL-11 in epithelial cancer cells [203]. CD8^+^ T cells express CXCR3 and are recruited into the tumor by the CXCR3 ligands CXCL-9, CXCL-10, CXCL-11. The chemokines CXCL-9, 10 and 11 not only show angiostatic effects but are also important factors for the recruitment and activation of T helper 1 cells, which express the corresponding receptor CXCR3 [203]. CTLs, therefore, have a great impact on the survival of CRC patients [202]. Patients with MSI status show much higher CTL infiltration and a better prognosis than MSS patients [204].

CD4^+^ T helper cells (T_H_ cells) differentiate into several subsets with divergent cytokine secretion and functions. T_H_ cells modulate immune responses by activating or suppressing activities of immune cells such as macrophages, B cells and CTLs. CD4^+^ T_H_ cells are divided by the ability to produce one or more signature cytokines and to express a lineage-specific transcription factor [205].

T_H_1 cells produce IFN-γ and TNF and express the Tbet transcription factor and the chemokine receptor CXCR3. Differentiation of naïve T_H_ cells occurs in response to viruses and intracellular bacteria. After activation and differentiation, T_H_1 cells express the pro-inflammatory cytokines TNF and IFN-*γ*, which activate CTLs to kill infected cells, help B cells to present antigens and to activate CD8^+^ T cells more efficiently, and stimulate macrophages to phagocyte dead cells and debris. In CRC, the infiltration of T_H_1 cells is positively correlated with good clinical outcome [44]. 

T_H_2 cells produce IL-4, IL-5 and IL-13 and express the transcription factor GATA-3 in response to extracellular pathogens. T_H_2 cells express the chemokine receptors CCR3, CCR4 and CCR10 that bind various CCL chemokines [206]. T_H_2 lymphocytes polarize macrophages toward a M2 phenotype, thus, acting as tumor-promoting players. IL-4 promotes tumor proliferation and increases the production of ROS. In experimental mouse models of CRC, IL-4 and IL-4R-deficient mice develop fewer tumors than the control animals [207]. This is a contradictory result as in CRC patients, the type 2 signature with IL-4, IL-5 and IL-13 has no prognostic advantage [112].

T_H_9 lymphocytes produce IL-9 and express PU.1. The role of T_H_9 cells in CRC is not clear as IL-9 has a strong inflammatory activity in experimental colitis, leading to colitis-associated cancer [208]. The expression of IL-9, however, was significantly lower in patients with CRC than in the control tissue and correlated with staging and prognosis [209]. 

T_H_17 lymphocytes produce IL-17A, IL-17F and IL-22 and express the transcription factor ROR*γ*t. T_H_17 lymphocytes express the chemokine receptors CCR6 and CXCR4 [43]. As explained in the previous paragraph, T_H_17 cells are important for the protection against extracellular bacterial infection. In a mouse model of colorectal cancer, the inhibition of IL-17 leads to significantly reduced tumorigenesis [109]. IL-17A induces the production of growth factors that stimulate the proliferation of myeloid cells, especially neutrophils. At the same time, IL-17A induces the production of CXCL-1 and CXCL-5, leading to the recruitment of the expanded myeloid cells into the tumor where they help to establish a tumor promoting microenvironment. Additionally, IL-17 promotes the production of IL-6 and TNF. IL-6, TNF and IL-17A promote the growth of CRC cells via STAT3 and NF-*κ*B [210]. The cytokine TGF-*β* induces the production of IL-22 in IL-17-positive T_H_17 cells and, subsequently, tumor progression [211]. T_H_17 cells occur much more frequently in MMR proficient CRC tumors than in MMR-deficient tumors [212].

Regulatory T cells produce TGF-*β*, IL-10 and IL-35 and express the transcription factor forkhead box P3 (FOXP3) and the chemokine receptors CCR4, CCR5, CCR10 and CXCR3 [205,213]. The cytokine TGF-*β* induces in the TME the transcription factor FOXP3 and the differentiation of Tregs. In the TME, TAMs and MDSCs secrete CCL-17, CCL-22, CCL-5, CCL-6 or CCL-28 to recruit regulatory T cells. T regs show a high immune suppression capacity, employing more than a dozen suppression mechanisms. They can suppress immune cells by producing the cytokines IL-10, TGF-*β* or IL-35. In addition, they show suppressive activities by depleting soluble and membrane bound molecules needed for effector functions such as depletion of extracellular adenosine triphosphate (ATP) or the stripping of co-stimulatory molecules from the surface of DCs [214,215]. Although Tregs effectively suppress the activities of CTLs and NK cells, their role in sporadic cancer is still unclear. Some studies described a reduced overall survival in the presence of high frequencies of Tregs among tumor-infiltrating lymphocytes [213,216]. However, high levels of infiltrated Tregs were also associated with good prognosis [217,218].

Natural killer (NK) cells belong to the family of innate lymphoid cells. NK cells are capable of killing tumor cells without antigen presentation and release TNF and IFN-*γ*. In CRC, the infiltration of cytotoxic NK cells is associated with a better prognosis [219].

Chronic inflammation is characterized by mucosal infiltration of inflammatory immune cells that interact with the local microbiota and induce a pro-inflammatory milieu. 

Regulatory B cells (Bregs) are immune suppressive and reduce the tissue damaging T_H_1/T_H_17 response by secreting IL-10 and IgA antibodies and restore the cytokine-induced balance [220]. Several cytokines, including IL-6, IL-33 and IL-35, induce the transition of B cells to Bregs [221]. Breg cells suppress the anti-tumorigenic immune response and lead to tumor progression by the tumor-promoting cytokine IL-10 [222]. In CRC, this tissue preserving and T_H_1-reducing activity mediated by IL-10 is one of the escape mechanisms of colorectal cancer cells leading to excessive proliferation of the tumor.

Other central players in coordinating immune responses against invading pathogens are dendritic cells. DCs are a heterogeneous population of antigen presenting cells that scan the environment for danger signals and antigens. In homeostasis, immature DCs have the capacity to process and present antigens to primed T cells. To avoid tissue damage and exacerbating immune responses these immature DCs secrete, similar to regulatory B cells, the immune-suppressive cytokines IL-10 and TGF-*β* and show low expression of co-stimulatory molecules. In the presence of PAMPs or DAMPs and inflammatory cytokines, the maturation process starts. Mature DCs upregulate co-stimulatory molecules and secrete the pro-inflammatory cytokines IL-12, IL-6, TNF, IL-1*β* that are required for priming, activation and proliferation of T cells. Several tumor-derived cytokines such as TGF-*β*, TNF, IL-6 or IL-10 are able to prevent the maturation of DCs, the infiltration of mature DCs, the presentation of antigens and the activation of T and NK cells [223,224].

The CMS classification made clear that CRC is a very heterogenous disease, especially regarding the tumor microenvironment. As an effective anti-tumorigenic response depends on infiltrating effector immune cells of the innate and adaptive immune system, chemokines that facilitate the recruitment of these cells are of great importance. In the CMS2 and the CMS3 subtypes, there is hardly any immune cell infiltration; therefore, these subtypes show poor anti-tumor immunity. One good example for the pivotal role of some chemokines is the expression of CXCR3 chemokines in myeloid cells. The pro-inflammatory cytokine IFN-*γ* induces CXCR3 chemokines, e.g., CXCL-9 and CXCL-10, as part of the interferon-stimulated gene cluster that then attracts T cells into the tumor [225]. However, as shown by CMS4, the infiltration of immune cells is not enough to fight the tumor. Cytokines within the TME determine the balance between tumor-promoting and tumor-destructive features of the immune cell infiltrate and the cells of the microenvironment (Figure 4). Cytokine and chemokine networks that are shaped by inflammatory processes regulate this balance. In the context of CRC, this is further complicated by the genotype of the tumor. Both MMR-deficient and -proficient CRCs show strong stromal remodeling with reduced amounts of fibroblasts compared with healthy colon samples. Both CRC types are characterized by inflammatory interaction networks of malignant epithelial cells, monocytes, CAFs and TANs at the luminal margin of the tumors. In both CRC subtypes, the pro-inflammatory cytokines IL1-*β*, IL-6 and TNF upregulate the chemokines CXCL-1/2/3/5/6/8 in CAFs, monocytes and cancer cells; this attracts CXCR1/2-positive neutrophils to the tumor. MMR-deficient CRCs respond to immune checkpoint therapy, whereas MMR-proficient CRCs are unresponsive to immunotherapy. The difference between both tumor types are foci in the MMR-deficient tumors with IFN-*γ*-positive and CXCL-13-positive T cells that form positive feedback loops to attract more activated T cells [226].

### 2.3. Cytokines in Therapy-Induced Inflammation

In recent years, therapies for CRC were based on targeting rapidly proliferating cancer cells by chemotherapeutic drugs and radiotherapy after surgery. All these therapeutic options are successful in early stages; in the case of metastatic CRC they are no longer effective, as seen by the 5-year survival rate of only 15% [227]. This poor treatment efficiency is partly due to drug resistance mediated by genetic and epigenetic changes in cancer cells [227]. However, several studies have shown that cytokines and the therapy-induced inflammation within the tumor play a substantial role that influences the balance of a tumor-suppressing TME and a tumor-promoting TME and the clinical outcome of therapies [26,228].

Chemotherapeutic drugs not only kill proliferating cells but influence the cytokine milieu within the TME. In general, chemotherapeutics are classified according to their mechanisms of killing proliferating cells into:

(I) alkylating agents that elicit DNA crosslinks and destabilize DNA during replication, such as cyclophosphamide and oxaliplatin.
(II) antimetabolites that disrupt DNA and RNA synthesis, such as 5-fluorouracil and gemcitabine
(III) topoisomerase inhibitors that interfere with DNA unwinding processes, such as irinotecan
(IV) microtubule poisons that inhibit tubulin polymerization and depolimerization such as paclitaxel
(V) antibiotics that kill via excessive ROS production and DNA intercalation, such as anthracyclines and bleomycin

All these chemotherapeutic drugs do not only kill cells but also have immunogenic effects by modulating lymphocytes and sometimes converting a tumor-promoting into a more tumor-suppressive TME. For example, oxaliplatin treatment increases the number of CD8^+^ CTLs and reduces the number of Tregs in CRC patients [229]. Interestingly, cisplatin, a chemically similar chemotherapeutic drug also used to treat CRC induces inflammation that enhances TNF-mediated angiogenesis, metastasis and failure of therapy [230]. Paclitaxel, a microtubule poison, induces apoptosis of cancer cells and activates IL-1*β*, IL-8, IL-6, thus, enhancing pro-tumorigenic inflammation [229].

Chemotherapy targets cells that are proliferating but CRC, similar to other cancer entities, is a heterogeneous disease with a small subpopulation of quiescent cancer cells with a stem-cell phenotype. These cancer stem cells (CSCs) have the ability to renew themselves as well as to differentiate and dedifferentiate. CSCs have long cell-cycle times, and therefore, chemotherapeutic drugs cannot kill them efficiently. There are very few therapeutic options; the most promising one is to target CRC-typical pathways such as Wnt/*β*-catenin, Notch, Hedgehog, NF-*κ*B, Janus kinase/STAT, peroxisome proliferator-activated receptor (PPAR), PI3K/Akt/mTOR and TGF-*β* pathways (for a detailed review see [231]). In CRC, there are several clinical studies that target these fundamental pathways, most of them in combination with chemotherapy. Among them, napabucasin, a STAT3 inhibitor in combination with FOLFIRI chemotherapy has completed phase III (CanStem303C; NCT02753127).

Cytotoxic therapies such as chemotherapies or radiotherapies elicit cell death of tumor cells and induce the secretion of DAMPs from dying cells [232]. DAMPs activate neutrophils to release IL-1 cytokines and chemokines. As described in the previous paragraph, neutrophils in CRC can promote the growth of cancer cells by regulating the innate and adaptive immune system and inducing angiogenesis [191] or decrease tumor growth through their cytotoxic capacity [233]. TANs can differentiate into a tumor-promoting N2 phenotype [191] or into N1 neutrophils with an anti-tumorigenic phenotype [192] depending on the cytokines within the microenvironment. For example, TGF-*β* polarizes toward a N2 phenotype whereas IFN-*β* toward a N1 phenotype.

DAMPs can activate CAFs and endothelial cells that express pro-inflammatory cytokines such as IL-6 and IL-8. Both cytokines activate immuno-suppressive cells such as TAMs and MDSCs within the tumor microenvironment, thus, promoting cancer immune evasion [228]. However, irradiation of a tumor that contains IL-1-secreting inflammatory CAFs can not only diminish the tumor-eradicating effect of radiotherapy but can lead to advanced cancer growth. The mechanism behind this astonishing effect is the IL-1-dependent induction of senescence in the inflammatory CAFs by radiation [194]. Senescent cells, although cell-cycle arrested, secrete various cytokines, growth factors and metalloproteinases, the so called senescence-associated secretory phenotype (SASP). The SASP can lead to advanced tumor growth, therapy resistance and senescence induction of tumor-infiltrating immune cells [234,235,236].

Radiation also induces DNA damage in the irradiated cells. If the DNA damage repair system cannot repair the damage, the irradiated cell becomes necrotic or apoptotic or senescent, depending on the cell type [237]. For example, CAFs, TAMs and mature DCs show radioresistance, whereas endothelial cells and NK cells are radiosensitive. DNA damage can elicit the same effects as viral infection with the accumulation of cytosolic DNA or RNA. Cytosolic DNA and RNA activate the cyclic GMP-AMP synthase (cGAS)-stimulator of IFN genes (STING) pathway, and the cells begin to secrete type I IFNs, TNF and IL-1 [237]. This activates the NF-*κ*B signaling pathway in the irradiated TME with the production of IL-6, TNF, IL-8 and CXCL-10. Dying cancer cells secrete several DAMPs such as ATP, a “find-me” signal for DCs [238]; calreticulin; high mobility group box 1 (HMGB1) or type I IFNs [239]. In the TME, immature DCs ingest DNA and RNA via phagocytosis, and this activates the cGAS-STING pathway and the production of type I IFNs. Type I IFNs bind to the receptor in an autocrine or paracrine manner and facilitate the maturation of CDs [240]. Together with the enhanced secretion of neo-antigens from dying tumor cells, activated DCs promote the activation of CD4^+^ and CD8^+^ T cells, monocytes and macrophages [241,242,243], rendering the pro-tumorigenic TME into a more anti-tumorigenic TME. Radiation can exhibit more tumor-suppressing effects as radiation induces secretion of tumor cell intrinsic CXCL-16 that recruits T_H_1 and CD8^+^ T cells to the tumor [239,244].

Surgical tumor resection can induce immunosuppressive activities by inducing pro-inflammatory and pro-tumorigenic cytokines such as IL-1*β*, IL-6, IL-10 CCL-2 and TGF-*β*, which recruit immunosuppressive cells such as MDSCs [245,246]. Tumor resection in CRC patients reduced the number of NK cells and other lymphocytes with anti-tumor activities [228,247].

## 3. Cytokine-Mediated Therapeutic Options and New Treatment Strategies

**Figure 5:**
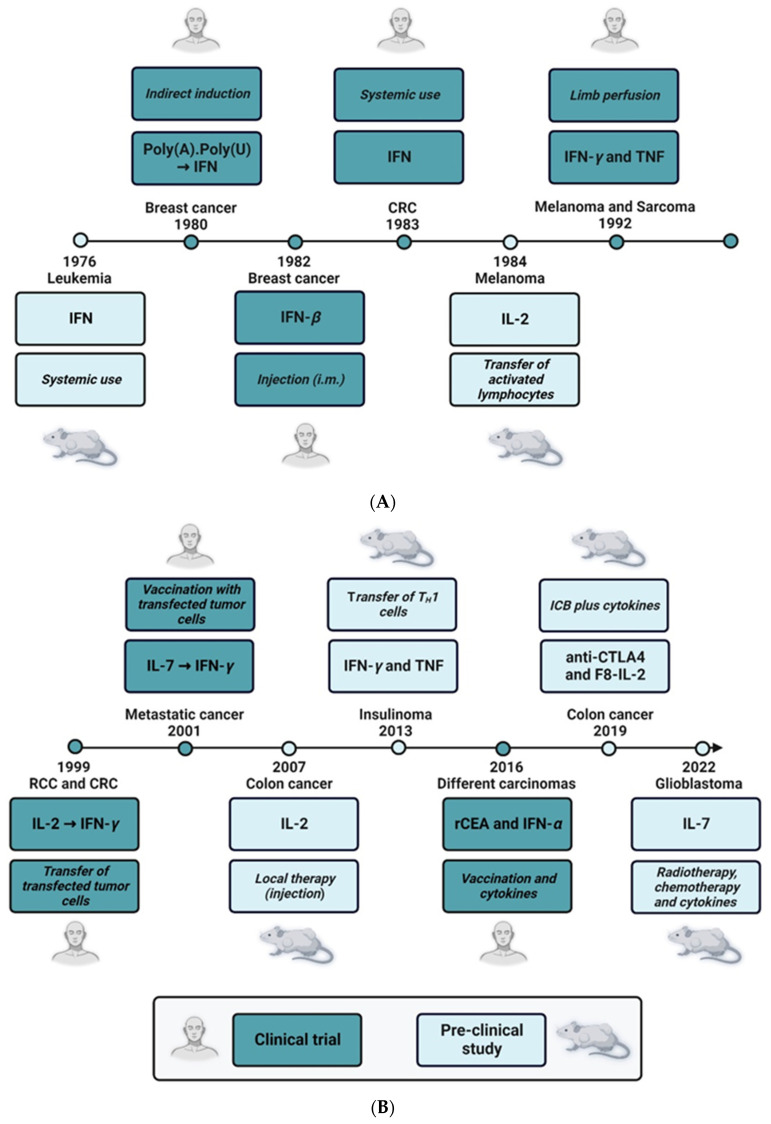
Timeline of cytokine-related cancer therapies. (**A**) 1976–1992. (**B**) 1999–2022. The time bar depicts the timely order of pre-clinical (light blue boxes) or clinical (dark blue boxes) therapeutic use of different cytokines, mainly interferons, or cytokines in combination therapy. The study designs, the administration routes, the use of genetically modified cells, the identity of the cytokines or therapeutic agents are stated in the respective boxes, and the targeted cancer types are stated either directly above or below the year of publication of the study. For references of the pre-clinical and clinical studies see in-text citations of chapter 3.

As described in detail above, the etiology of CRC comprises multiple factors, e.g., genetic predisposition and mutations [248], microbiota [249], diet and other environmental factors such as chronic inflammation [250]. Recently, different genetic factors that may serve as diagnostic and therapeutic biomarkers in CRC have been listed in detail [248]. The related gene products are mainly proteins that are known to regulate basic cellular pathways such as cell proliferation, cell cycle and apoptosis, and are, thus, well-known players in other tumor entities as well: APC, beta-catenin, carcinoembryonic antigen (CEA), insulin-like growth factor-1 receptor (IGF1R), PI3 kinase, TP53, etc. Surprisingly, with the exception of TGF-*β* and CXCR4, known direct regulators of the immune system do not appear on this list. Thus, especially during the development of CRC, the immune response does not seem to be basically disturbed by severe mutations. As outlined in chapter 2, however, inflammation and inflammation-related pathways play important roles in the tumorigenesis of CRC (for review see also [250]). Severe inflammation can be interpreted as a hyperactivation of the immune system, including the enhanced activity of specific immune cells, and chronic inflammation is an activation process of the immune system that fails to be terminated in due time [251]. The involvement of the immune response in CRC is, therefore, not caused by functional mutations of specific immune factors but rather by inaccurate quantitative regulation or by a wrong timing of the immune response. Cytokines are the main operators of the communication network between the different immune cells. They regulate the proliferative state and the effector functions of the immune cells, i.e., T cells, B cells, NK cells, macrophages, DCs and others, thereby indirectly repelling and/or fostering cancer development. In addition, they may also exert a direct therapeutic effect by the inhibition of tumor cell proliferation or induction of apoptosis. Thus, cytokines and related factors are possible drug candidates as well as putative drug targets (e.g., for indirect induction or stabilization of an immune response). Indeed, cytokines have now been known for their antitumoral efficacy for more than five decades (for the timeline see Figure 5) with IFNs and ILs being the most prominent representatives of this new class of biologicals.

In 1976, a pre-clinical study demonstrated that treatment of owl monkeys with human IFN had a positive antileukemic effect in Herpesvirus saimiri-induced disease [252]. Only 4 years later, adjuvant immunotherapy with polyadenylic-polyuridylic acid (PolyA.PolyU), a formulation that indirectly leads to increased IFN levels [253], was tested in a randomized trial on 300 patients with operable breast cancer. Here, the treatment of patients with PolyA.PolyU in the study group (155 patients) significantly increased the overall survival as compared with the control group (145 patients), who received normal saline [254]. These promising data were confirmed in an early report using partially purified human IFN-*β*. The therapeutic agent was administered to six patients with metastatic breast carcinoma by the intramuscular route, and objective antitumor effects were observed in half of the patients [255]. However, there were also significant systemic side effects, e.g., fatigue, fever, pruritus and nausea. This already pointed to the main drawback of therapeutic use of IFN, namely, the difficulty of systemic application. In the case of metastatic colon carcinoma, the results of the systemic use of IFNs were even more discouraging, and treatment of patients suffering from colon cancer with human lymphoblastoid IFN failed to demonstrate significant regressions of malignant lesions [256]. As a consequence, other routes of administration were tested. In 1992, the new technique of limb perfusion was introduced to treat melanoma and sarcoma patients with a combination of IFN-*γ* and TNF. In depth analysis of the phase II study suggested that the combination of recombinant IFN-*γ*, recombinant TNF and the drug melphalan achieved high therapeutic efficacy with minimal toxicity [257]. Mechanistic follow-up papers showed that the therapeutic effect of the cytokine cocktail may be due to its anti-vascular activity. In the same study, detachment and apoptosis of angiogenic endothelial cells was demonstrated in vivo in melanoma metastases of patients treated with IFN-*γ* and TNF [258]. Another possibility to bring IFNs to the right place where they can exert their antitumoral effects is targeted delivery by tumor-specific T_H_1 cells. The IFN-*γ*- and TNF-producing T_H_1 cells migrate to the transformed tissue, thereby surrounding the tumor lesions and secreting the soluble cytokines (see also Figure 5). In this study, T_H_1 cells were able to stop insulinoma growth in RIP-Tag2 mice by IFN-*γ*- and TNF-dependent senescence induction [259]. Interestingly, IFNs also had a come-back in the therapy of CEA-expressing carcinoma. It was shown recently that prime-boost vaccination with recombinant vaccinia(V)-CEA(6D)-TRICOM (which is a triad of co-stimulatory molecules B7.1, intercellular adhesion molecule 1 (ICAM-1) and lymphocyte function-associated antigen 3 (LFA-3)) together with the administration of granulocyte-macrophage colony-stimulating factor (GM-CSF) and IFN-*α* demonstrated good efficacy in CEA-expressing cancers. Here, IFN-*α* was clearly associated with improved survival [260].

The therapeutic use of IL-2 was first published in 1984, nearly one decade after the first use of IFN (Figure 5). IL-2 is a well-known stimulator of in vitro T lymphocyte proliferation [261]. The antitumor effect of this cytokine is due to an indirect mechanism presumably by stimulating the T cell-mediated antitumor defense. Thus, IL-2 was used to activate syngeneic lymphocytes in vitro. These lymphokine-activated killer (LAK) cells were then adoptively transferred in a murine B16 metastasis model leading to decreased numbers of lung nodules and improved survival of the mice [262]. A similar approach was tested in renal and colon cancer patients some 15 years later. In a phase I clinical protocol, autologous cytokine-induced killer cells were generated from peripheral blood obtained from patients with metastatic cancer. These cells were transfected with the IL-2 gene via electroporation and transferred by repeated intravenous infusions. With the exception of WHO grade 2 fever, the treatment schedule did not show severe side effects. However, with only one complete remission out of 10 patients in the treatment group, the clinical outcome was negligible [263]. Nevertheless, this study paved the way for other transfection experiments, and only two years later, a vaccination protocol using autologous IL-7-transfected tumor cells was tested in a phase I/II trial. The results of this study were much more promising as half of the treated patients showed at least a partial response or stable disease without adverse effects [264].

In 2007, local therapy with IL-2 was demonstrated to be very effective in the treatment of different forms of cancer. Syngenic rats were inoculated with neoplastic cells and then either injected with IL-2 in bovine serum albumin (BSA) solution or with BSA alone at the site of inoculation. After three weeks, the volume and weight of the neoplastic tissue, as well as the mitotic index of the lesions were significantly reduced in the treatment group [265]. This approach was refined recently. Here, recombinant human IL-2 was combined with immune checkpoint inhibitors. In order to increase the therapeutic window, F8-IL-2 was used, an antibody-IL-2 fusion protein which selectively localizes to the tumor site. Interestingly, the combination of F8-IL-2 with cytotoxic T-lymphocyte-associated protein 4 (CTLA-4) blockade lead to an increased progression-free survival of colon carcinoma-bearing mice [266]. The treatment regimen also demonstrated protective immunity against subsequent tumor rechallenges. Recently, a long-acting recombinant IL-7, NT-I7, in combination with radiotherapy and temozolomide, a cytostatic drug, was tested in a mouse glioma model. The results of this study demonstrated an improved survival of the mice of the treatment group, an effect that was dependent on IFN-*γ* and CD8^+^ cells [267]. The use of NT-I7 is currently evaluated in patients with high-grade gliomas (ongoing clinical trial NCT03687957).

As outlined above, intensive pre-clinical and clinical research led to a significant number of cytokine-mediated treatment options. This is reflected by the high number of clinical trials targeting different signaling molecules of the immune system (for a detailed overview see [187]). Concerning CRC, the following clinical trials are of special interest: (i) NCT04599140 with the immune target CXCR1/2 (Start 2020); (ii) NCT02466906 with the immune target GM-CSF (Start 2015); (iii) NCT00030342 with the immune targets GM-CSF and IFN-*α* (Start 2001); (iv) NCT00002796 with the immune target IFN-*γ* (Start 1997); (v) NCT04426669 with the immune target IL-2 (Start 2020); (vi) NCT00072098 with the immune target IL-12 (Start 2003); (vii) NCT03436563 with the immune target TGF-*β* (Start 2018); and (viii) NCT04708470 with the immune targets TGF-*β* and IL-12 (Start 2021). 

## 4. Conclusions

The etiology of colorectal cancer (CRC) comprises multiple factors, including genetic and environmental factors. Cancer cells communicate with neighboring cells via soluble factors, e.g., cytokines or chemokines, to generate a favorable tumor microenvironment (TME). Here, we thoroughly discuss the cytokine networks, including the cytokine-producing cells of the TME, which can be found in the different stages of CRC development. Together, pre-clinical and clinical research resulted in the therapeutic use of cytokines in tumor therapy, which now has a long-standing tradition of over 40 years also in the therapy of CRC. As cytokines are endogenous substances, however, their systemic use clearly shows limitations, and cytokine-related side effects are frequently observed. In the future, the main focus of clinical research will be on combination therapies using (targeted) cytokines together with other established therapies such as immune checkpoint blockade, adoptive immune cell transfer, chemotherapy or radiotherapy.

## Abbreviations

APC	Adenomatous Polyposis Coli
ATP	Adenosine Triphosphate
BRAF	B-Raf and v-Raf Murine Sarcoma Viral Oncogene Homolog B
Breg	Regulatory B Cell
BSA	Bovine Serum Albumin
CAC	Colitis-Associated Cancer
CAF	Cancer-Associated Fibroblast
CCL2	CC-Chemokine Ligand 2
CCR6	CC Chemokine Receptor 6
CD4	Cluster of Differentiation Antigen 4
CEA	Carcinoembryonic Antigen
CIMP	CpG Island Methylator Phenotype
CIN	Chromosomal Instability
CMS	Consensus Molecular Subtype
CRC	Colorectal Cancer
CTL	Cytotoxic T Lymphocyte
CTLA-4	Cytotoxic T-Lymphocyte-Associated Protein 4
CTNNB1	Catenin Beta 1
CXCL-12	C-X-C Chemokine Ligand 12
CXCR4	C-X-C Chemokine Receptor Type 4
DAMP	Damage-Associated Molecular Pattern
DC	Dendritic Cell
DCC	Deleted in Colorectal Carcinoma
DNA	Deoxyribonucleic Acid
EMT	Epithelial–Mesenchymal Transition
FOXP3	Forkhead Box P3
GM-CSF	Granulocyte-Macrophage Colony-Stimulating Factor
gp130	Glycoprotein 130
HRAS	Harvey Rat Sarcoma Virus
IBD	Inflammatory Bowel Disease
ICAM-1	Intercellular Adhesion Molecule 1
IEC	Intestinal Epithelial Cell
IEL	Intraepithelial Lymphocyte
IFN-*γ*	Interferon-Gamma
IFNAR1	Interferon Alpha and Beta Receptor Subunit 1
IGF1R	Insulin-Like Growth Factor-1 Receptor
IL-17	Interleukin-17
IL-1R1	Interleukin 1 Receptor Type 1
KRAS	Kirsten Rat Sarcoma Virus Oncogene
LAK	Lymphokine-Activated Killer Cell
LFA-3	Lymphocyte Function-Associated Antigen 3
LOH	Loss Of Heterozygosity
LPS	Lipopolysaccharide
M2	Macrophage Type 2
MAP	Mitogen-Activated Protein
MCC	Mutated in Colorectal Cancer
MDSC	Myeloid-Derived Suppressor Cell
MLH1	MutL Homolog 1
MMR	Mismatch Repair
MSH6	MutS Homolog 6
MSI	Microsatellite Instability
N1	Neutrophil Type 1
NFkB	Nuclear Factor Kappa-B
NK	Natural Killer Cell
NRAS	Neuroblastoma Rat Sarcoma Virus
OSMRb	Oncostatin-M-Specific Receptor b
PAMPs	Pathogen-Associated Molecular Patterns
PI3K	Phosphatidylinositol 3-Kinase
PIK3CA	Phosphatidylinositol-4,5-Bisphosphate 3-Kinase, Catalytic Subunit Alpha
PMS1	Protein Homolog 1
Poly(A).Poly(U)	Polyadenylic-Polyuridylic Acid
RCT	Randomized Controled Trial
RIP-Tag2	Rat Insuline Promotor T Antigen 2
ROS	Reactive Oxygen Species
SCFAs	Short-Chain Fatty Acids
SDF-1	Stromal Cell-Derived Factor 1
STAT3	Signal Transducer and Activator of Transcription 3
TAM	Tumor-Associated Macrophage
TAN	Tumor-Associated Neutrophil
TGF-*β*	Transforming Growth Factor Beta
TGFBR1	Transforming Growth Factor-Beta Receptor 1
T_H_1	T Helper 1 Cell
TIL	Tumor-Infiltrating Lymphocyte
TIM	Tumor-Infiltrating Myeloid Cell
TLR	Toll-Like Receptor
TME	Tumor Microenvironment
TNF	Tumor Necrosis Factor
TNFR1	Tumor Necrosis Factor Receptor 1
Treg	T Regulatory Cell
WHO	World Health Organization
WNT	Wingless Pathway

## References

[1] Xi Y., Xu P. (2021). Global colorectal cancer burden in 2020 and projections to 2040. Transl. Oncol..

[2] Keum N., Giovannucci E. (2019). Global burden of colorectal cancer: Emerging trends, risk factors and prevention strategies. Nat. Rev. Gastroenterol. Hepatol..

[3] Campos F., Figueiredo M.N., Monteiro M., Nahas S.C., Cecconello I. (2017). Incidence of colorectal cancer in young patients. Rev. Col. Bras. Cir..

[4] El Bali M., Bakkach J., Bennani Mechita M. (2021). Colorectal Cancer: From Genetic Landscape to Targeted Therapy. J. Oncol..

[5] Lynch H.T., de la Chapelle A. (2003). Hereditary colorectal cancer. N. Engl. J. Med..

[6] Power D.G., Gloglowski E., Lipkin S.M. (2010). Clinical genetics of hereditary colorectal cancer. Hematol. Oncol. Clin. N. Am..

[7] Stoffel E.M., Kastrinos F. (2014). Familial colorectal cancer, beyond Lynch syndrome. Clin. Gastroenterol. Hepatol..

[8] Fearon E.R., Vogelstein B. (1990). A genetic model for colorectal tumorigenesis. Cell.

[9] Powell S.M., Zilz N., Beazer-Barclay Y., Bryan T.M., Hamilton S.R., Thibodeau S.N., Vogelstein B., Kinzler K.W. (1992). APC mutations occur early during colorectal tumorigenesis. Nature.

[10] Karapetis C.S., Khambata-Ford S., Jonker D.J., O’Callaghan C.J., Tu D., Tebbutt N.C., Simes R.J., Chalchal H., Shapiro J.D., Robitaille S. (2008). K-ras mutations and benefit from cetuximab in advanced colorectal cancer. N. Engl. J. Med..

[11] Herr R., Kohler M., Andrlova H., Weinberg F., Moller Y., Halbach S., Lutz L., Mastroianni J., Klose M., Bittermann N. (2015). B-Raf inhibitors induce epithelial differentiation in BRAF-mutant colorectal cancer cells. Cancer Res..

[12] Hall D.C.N., Benndorf R.A. (2022). Aspirin sensitivity of PIK3CA-mutated Colorectal Cancer: Potential mechanisms revisited. Cell Mol. Life Sci..

[13] Fang J.Y., Richardson B.C. (2005). The MAPK signalling pathways and colorectal cancer. Lancet Oncol..

[14] Duan S., Huang W., Liu X., Liu X., Chen N., Xu Q., Hu Y., Song W., Zhou J. (2018). IMPDH2 promotes colorectal cancer progression through activation of the PI3K/AKT/mTOR and PI3K/AKT/FOXO1 signaling pathways. J. Exp. Clin. Cancer Res..

[15] Woodford-Richens K.L., Rowan A.J., Gorman P., Halford S., Bicknell D.C., Wasan H.S., Roylance R.R., Bodmer W.F., Tomlinson I.P. (2001). SMAD4 mutations in colorectal cancer probably occur before chromosomal instability, but after divergence of the microsatellite instability pathway. Proc. Natl. Acad. Sci. USA.

[16] Tarafa G., Villanueva A., Farre L., Rodriguez J., Musulen E., Reyes G., Seminago R., Olmedo E., Paules A.B., Peinado M.A. (2000). DCC and SMAD4 alterations in human colorectal and pancreatic tumor dissemination. Oncogene.

[17] Nakayama M., Wang D., Kok S.Y., Oshima H., Oshima M. (2022). Genetic Alterations and Microenvironment that Drive Malignant Progression of Colorectal Cancer: Lessons from Mouse and Organoid Models. J. Cancer Prev..

[18] Li H., Zhang J., Tong J.H.M., Chan A.W.H., Yu J., Kang W., To K.F. (2019). Targeting the Oncogenic p53 Mutants in Colorectal Cancer and Other Solid Tumors. Int. J. Mol. Sci..

[19] Kim H., Jen J., Vogelstein B., Hamilton S.R. (1994). Clinical and pathological characteristics of sporadic colorectal carcinomas with DNA replication errors in microsatellite sequences. Am. J. Pathol..

[20] Boland C.R., Goel A. (2010). Microsatellite instability in colorectal cancer. Gastroenterology.

[21] Wong J.J., Hawkins N.J., Ward R.L. (2007). Colorectal cancer: A model for epigenetic tumorigenesis. Gut.

[22] Fatemi N., Tierling S., Es H.A., Varkiani M., Mojarad E.N., Aghdaei H.A., Walter J., Totonchi M. (2022). DNA methylation biomarkers in colorectal cancer: Clinical applications for precision medicine. Int. J. Cancer.

[23] Guinney J., Dienstmann R., Wang X., de Reynies A., Schlicker A., Soneson C., Marisa L., Roepman P., Nyamundanda G., Angelino P. (2015). The consensus molecular subtypes of colorectal cancer. Nat. Med..

[24] Becht E., de Reynies A., Giraldo N.A., Pilati C., Buttard B., Lacroix L., Selves J., Sautes-Fridman C., Laurent-Puig P., Fridman W.H. (2016). Immune and Stromal Classification of Colorectal Cancer Is Associated with Molecular Subtypes and Relevant for Precision Immunotherapy. Clin. Cancer Res..

[25] Galon J., Costes A., Sanchez-Cabo F., Kirilovsky A., Mlecnik B., Lagorce-Pages C., Tosolini M., Camus M., Berger A., Wind P. (2006). Type, density, and location of immune cells within human colorectal tumors predict clinical outcome. Science.

[26] Fridman W.H., Zitvogel L., Sautes-Fridman C., Kroemer G. (2017). The immune contexture in cancer prognosis and treatment. Nat. Rev. Clin. Oncol..

[27] Lan T., Chen L., Wei X. (2021). Inflammatory Cytokines in Cancer: Comprehensive Understanding and Clinical Progress in Gene Therapy. Cells.

[28] Balkwill F.R., Mantovani A. (2012). Cancer-related inflammation: Common themes and therapeutic opportunities. Semin. Cancer Biol..

[29] Greten F.R., Grivennikov S.I. (2019). Inflammation and Cancer: Triggers, Mechanisms, and Consequences. Immunity.

[30] Grivennikov S.I., Cominelli F. (2016). Colitis-Associated and Sporadic Colon Cancers: Different Diseases, Different Mutations?. Gastroenterology.

[31] Robles A.I., Traverso G., Zhang M., Roberts N.J., Khan M.A., Joseph C., Lauwers G.Y., Selaru F.M., Popoli M., Pittman M.E. (2016). Whole-Exome Sequencing Analyses of Inflammatory Bowel Disease-Associated Colorectal Cancers. Gastroenterology.

[32] Joanito I., Wirapati P., Zhao N., Nawaz Z., Yeo G., Lee F., Eng C.L.P., Macalinao D.C., Kahraman M., Srinivasan H. (2022). Single-cell and bulk transcriptome sequencing identifies two epithelial tumor cell states and refines the consensus molecular classification of colorectal cancer. Nat. Genet..

[33] Briukhovetska D., Dorr J., Endres S., Libby P., Dinarello C.A., Kobold S. (2021). Interleukins in cancer: From biology to therapy. Nat. Rev. Cancer.

[34] Li J., Huang L., Zhao H., Yan Y., Lu J. (2020). The Role of Interleukins in Colorectal Cancer. Int. J. Biol. Sci..

[35] Leonard W.J., Lin J.X., O’Shea J.J. (2019). The gamma(c) Family of Cytokines: Basic Biology to Therapeutic Ramifications. Immunity.

[36] Bhat A.A., Nisar S., Singh M., Ashraf B., Masoodi T., Prasad C.P., Sharma A., Maacha S., Karedath T., Hashem S. (2022). Cytokine- and chemokine-induced inflammatory colorectal tumor microenvironment: Emerging avenue for targeted therapy. Cancer Commun..

[37] Rebe C., Ghiringhelli F. (2020). Interleukin-1beta and Cancer. Cancers.

[38] Fridman W.H., Miller I., Sautes-Fridman C., Byrne A.T. (2020). Therapeutic Targeting of the Colorectal Tumor Stroma. Gastroenterology.

[39] Boukhaled G.M., Harding S., Brooks D.G. (2021). Opposing Roles of Type I Interferons in Cancer Immunity. Annu. Rev. Pathol..

[40] Gocher A.M., Workman C.J., Vignali D.A.A. (2022). Interferon-gamma: Teammate or opponent in the tumour microenvironment?. Nat. Rev. Immunol..

[41] Galon J., Lanzi A. (2020). Immunoscore and its introduction in clinical practice. Q. J. Nucl. Med. Mol. Imaging.

[42] Korbecki J., Kojder K., Siminska D., Bohatyrewicz R., Gutowska I., Chlubek D., Baranowska-Bosiacka I. (2020). CC Chemokines in a Tumor: A Review of Pro-Cancer and Anti-Cancer Properties of the Ligands of Receptors CCR1, CCR2, CCR3, and CCR4. Int. J. Mol. Sci..

[43] Nagarsheth N., Wicha M.S., Zou W. (2017). Chemokines in the cancer microenvironment and their relevance in cancer immunotherapy. Nat. Rev. Immunol..

[44] Bruni D., Angell H.K., Galon J. (2020). The immune contexture and Immunoscore in cancer prognosis and therapeutic efficacy. Nat. Rev. Cancer.

[45] Zumwalt T.J., Arnold M., Goel A., Boland C.R. (2015). Active secretion of CXCL10 and CCL5 from colorectal cancer microenvironments associates with GranzymeB+ CD8+ T-cell infiltration. Oncotarget.

[46] Zou Q., Lei X., Xu A., Li Z., He Q., Huang X., Xu G., Tian F., Ding Y., Zhu W. (2022). Chemokines in progression, chemoresistance, diagnosis, and prognosis of colorectal cancer. Front. Immunol..

[47] Li B., Qi Z.P., He D.L., Chen Z.H., Liu J.Y., Wong M.W., Zhang J.W., Xu E.P., Shi Q., Cai S.L. (2021). NLRP7 deubiquitination by USP10 promotes tumor progression and tumor-associated macrophage polarization in colorectal cancer. J. Exp. Clin. Cancer Res..

[48] Do H.T.T., Lee C.H., Cho J. (2020). Chemokines and their Receptors: Multifaceted Roles in Cancer Progression and Potential Value as Cancer Prognostic Markers. Cancers.

[49] Khare T., Bissonnette M., Khare S. (2021). CXCL12-CXCR4/CXCR7 Axis in Colorectal Cancer: Therapeutic Target in Preclinical and Clinical Studies. Int. J. Mol. Sci..

[50] Strater J., Koretz K., Gunthert A.R., Moller P. (1995). In situ detection of enterocytic apoptosis in normal colonic mucosa and in familial adenomatous polyposis. Gut.

[51] Ngo P.A., Neurath M.F., Lopez-Posadas R. (2022). Impact of Epithelial Cell Shedding on Intestinal Homeostasis. Int. J. Mol. Sci..

[52] Scarpa M., Kessler S., Sadler T., West G., Homer C., McDonald C., de la Motte C., Fiocchi C., Stylianou E. (2015). The epithelial danger signal IL-1alpha is a potent activator of fibroblasts and reactivator of intestinal inflammation. Am. J. Pathol..

[53] Nunberg M., Werbner N., Neuman H., Bersudsky M., Braiman A., Ben-Shoshan M., Ben Izhak M., Louzoun Y., Apte R.N., Voronov E. (2018). Interleukin 1alpha-Deficient Mice Have an Altered Gut Microbiota Leading to Protection from Dextran Sodium Sulfate-Induced Colitis. mSystems.

[54] Menghini P., Corridoni D., Butto L.F., Osme A., Shivaswamy S., Lam M., Bamias G., Pizarro T.T., Rodriguez-Palacios A., Dinarello C.A. (2019). Neutralization of IL-1alpha ameliorates Crohn’s disease-like ileitis by functional alterations of the gut microbiome. Proc. Natl. Acad. Sci. USA.

[55] Impellizzeri D., Siracusa R., Cordaro M., Peritore A.F., Gugliandolo E., Mancuso G., Midiri A., Di Paola R., Cuzzocrea S. (2018). Therapeutic potential of dinitrobenzene sulfonic acid (DNBS)-induced colitis in mice by targeting IL-1beta and IL-18. Biochem. Pharmacol..

[56] Bersudsky M., Luski L., Fishman D., White R.M., Ziv-Sokolovskaya N., Dotan S., Rider P., Kaplanov I., Aychek T., Dinarello C.A. (2014). Non-redundant properties of IL-1alpha and IL-1beta during acute colon inflammation in mice. Gut.

[57] Matarazzo L., Hernandez Santana Y.E., Walsh P.T., Fallon P.G. (2022). The IL-1 cytokine family as custodians of barrier immunity. Cytokine.

[58] Coccia M., Harrison O.J., Schiering C., Asquith M.J., Becher B., Powrie F., Maloy K.J. (2012). IL-1beta mediates chronic intestinal inflammation by promoting the accumulation of IL-17A secreting innate lymphoid cells and CD4(+) Th17 cells. J. Exp. Med..

[59] Lopetuso L.R., Chowdhry S., Pizarro T.T. (2013). Opposing Functions of Classic and Novel IL-1 Family Members in Gut Health and Disease. Front. Immunol..

[60] Bradford E.M., Ryu S.H., Singh A.P., Lee G., Goretsky T., Sinh P., Williams D.B., Cloud A.L., Gounaris E., Patel V. (2017). Epithelial TNF Receptor Signaling Promotes Mucosal Repair in Inflammatory Bowel Disease. J. Immunol..

[61] Jeffery V., Goldson A.J., Dainty J.R., Chieppa M., Sobolewski A. (2017). IL-6 Signaling Regulates Small Intestinal Crypt Homeostasis. J. Immunol..

[62] Song Y., Gu H.D., He Y., Wang J.W. (2015). Role of IL-6 polymorphism on the development of cardiovascular events and coronary artery disease in patients receiving hemodialysis. Genet. Mol. Res..

[63] Lindemans C.A., Calafiore M., Mertelsmann A.M., O’Connor M.H., Dudakov J.A., Jenq R.R., Velardi E., Young L.F., Smith O.M., Lawrence G. (2015). Interleukin-22 promotes intestinal-stem-cell-mediated epithelial regeneration. Nature.

[64] Aparicio-Domingo P., Romera-Hernandez M., Karrich J.J., Cornelissen F., Papazian N., Lindenbergh-Kortleve D.J., Butler J.A., Boon L., Coles M.C., Samsom J.N. (2015). Type 3 innate lymphoid cells maintain intestinal epithelial stem cells after tissue damage. J. Exp. Med..

[65] Scheibe K., Backert I., Wirtz S., Hueber A., Schett G., Vieth M., Probst H.C., Bopp T., Neurath M.F., Neufert C. (2017). IL-36R signalling activates intestinal epithelial cells and fibroblasts and promotes mucosal healing in vivo. Gut.

[66] von Moltke J., Ji M., Liang H.E., Locksley R.M. (2016). Tuft-cell-derived IL-25 regulates an intestinal ILC2-epithelial response circuit. Nature.

[67] Mahapatro M., Erkert L., Becker C. (2021). Cytokine-Mediated Crosstalk between Immune Cells and Epithelial Cells in the Gut. Cells.

[68] Hirano T., Hirayama D., Wagatsuma K., Yamakawa T., Yokoyama Y., Nakase H. (2020). Immunological Mechanisms in Inflammation-Associated Colon Carcinogenesis. Int. J. Mol. Sci..

[69] Balkwill F., Mantovani A. (2001). Inflammation and cancer: Back to Virchow?. Lancet.

[70] Varga J., Greten F.R. (2017). Cell plasticity in epithelial homeostasis and tumorigenesis. Nat. Cell Biol..

[71] Ritter B., Greten F.R. (2019). Modulating inflammation for cancer therapy. J. Exp. Med..

[72] Miller A.K., Williams S.M. (2021). Helicobacter pylori infection causes both protective and deleterious effects in human health and disease. Genes Immun..

[73] Senchukova M.A., Tomchuk O., Shurygina E.I. (2021). Helicobacter pylori in gastric cancer: Features of infection and their correlations with long-term results of treatment. World J. Gastroenterol..

[74] Drewes J.L., Chen J., Markham N.O., Knippel R.J., Domingue J.C., Tam A.J., Chan J.L., Kim L., McMann M., Stevens C. (2022). Human Colon Cancer-Derived Clostridioides difficile Strains Drive Colonic Tumorigenesis in Mice. Cancer Discov..

[75] Coussens L.M., Werb Z. (2002). Inflammation and cancer. Nature.

[76] Hua X., Phipps A.I., Burnett-Hartman A.N., Adams S.V., Hardikar S., Cohen S.A., Kocarnik J.M., Ahnen D.J., Lindor N.M., Baron J.A. (2017). Timing of Aspirin and Other Nonsteroidal Anti-Inflammatory Drug Use Among Patients With Colorectal Cancer in Relation to Tumor Markers and Survival. J. Clin. Oncol..

[77] Elinav E., Strowig T., Kau A.L., Henao-Mejia J., Thaiss C.A., Booth C.J., Peaper D.R., Bertin J., Eisenbarth S.C., Gordon J.I. (2011). NLRP6 inflammasome regulates colonic microbial ecology and risk for colitis. Cell.

[78] Leppkes M., Neurath M.F. (2020). Cytokines in inflammatory bowel diseases—Update 2020. Pharmacol. Res..

[79] Sommer K., Wiendl M., Muller T.M., Heidbreder K., Voskens C., Neurath M.F., Zundler S. (2021). Intestinal Mucosal Wound Healing and Barrier Integrity in IBD-Crosstalk and Trafficking of Cellular Players. Front. Med..

[80] Chen B., Scurrah C.R., McKinley E.T., Simmons A.J., Ramirez-Solano M.A., Zhu X., Markham N.O., Heiser C.N., Vega P.N., Rolong A. (2021). Differential pre-malignant programs and microenvironment chart distinct paths to malignancy in human colorectal polyps. Cell.

[81] Martincorena I., Campbell P.J. (2015). Somatic mutation in cancer and normal cells. Science.

[82] Busque L., Patel J.P., Figueroa M.E., Vasanthakumar A., Provost S., Hamilou Z., Mollica L., Li J., Viale A., Heguy A. (2012). Recurrent somatic TET2 mutations in normal elderly individuals with clonal hematopoiesis. Nat. Genet..

[83] Xie M., Lu C., Wang J., McLellan M.D., Johnson K.J., Wendl M.C., McMichael J.F., Schmidt H.K., Yellapantula V., Miller C.A. (2014). Age-related mutations associated with clonal hematopoietic expansion and malignancies. Nat. Med..

[84] Lee-Six H., Olafsson S., Ellis P., Osborne R.J., Sanders M.A., Moore L., Georgakopoulos N., Torrente F., Noorani A., Goddard M. (2019). The landscape of somatic mutation in normal colorectal epithelial cells. Nature.

[85] Garlanda C., Mantovani A. (2021). Interleukin-1 in tumor progression, therapy, and prevention. Cancer Cell.

[86] Pastille E., Wasmer M.H., Adamczyk A., Vu V.P., Mager L.F., Phuong N.N.T., Palmieri V., Simillion C., Hansen W., Kasper S. (2019). The IL-33/ST2 pathway shapes the regulatory T cell phenotype to promote intestinal cancer. Mucosal Immunol..

[87] Bergmann H., Roth S., Pechloff K., Kiss E.A., Kuhn S., Heikenwalder M., Diefenbach A., Greten F.R., Ruland J. (2017). Card9-dependent IL-1beta regulates IL-22 production from group 3 innate lymphoid cells and promotes colitis-associated cancer. Eur. J. Immunol..

[88] Chen J., Gong C., Mao H., Li Z., Fang Z., Chen Q., Lin M., Jiang X., Hu Y., Wang W. (2018). E2F1/SP3/STAT6 axis is required for IL-4-induced epithelial-mesenchymal transition of colorectal cancer cells. Int. J. Oncol..

[89] Lin X., Wang S., Sun M., Zhang C., Wei C., Yang C., Dou R., Liu Q., Xiong B. (2019). miR-195-5p/NOTCH2-mediated EMT modulates IL-4 secretion in colorectal cancer to affect M2-like TAM polarization. J. Hematol. Oncol..

[90] Ohno Y., Kitamura H., Takahashi N., Ohtake J., Kaneumi S., Sumida K., Homma S., Kawamura H., Minagawa N., Shibasaki S. (2016). IL-6 down-regulates HLA class II expression and IL-12 production of human dendritic cells to impair activation of antigen-specific CD4(+) T cells. Cancer Immunol. Immunother..

[91] Zhou L., Ivanov I., Spolski R., Min R., Shenderov K., Egawa T., Levy D.E., Leonard W.J., Littman D.R. (2007). IL-6 programs T(H)-17 cell differentiation by promoting sequential engagement of the IL-21 and IL-23 pathways. Nat. Immunol..

[92] Hirano T. (2021). IL-6 in inflammation, autoimmunity and cancer. Int. Immunol..

[93] Heichler C., Scheibe K., Schmied A., Geppert C.I., Schmid B., Wirtz S., Thoma O.M., Kramer V., Waldner M.J., Buttner C. (2020). STAT3 activation through IL-6/IL-11 in cancer-associated fibroblasts promotes colorectal tumour development and correlates with poor prognosis. Gut.

[94] Grivennikov S.I. (2013). Inflammation and colorectal cancer: Colitis-associated neoplasia. Semin. Immunopathol..

[95] Grivennikov S.I. (2013). IL-11: A prominent pro-tumorigenic member of the IL-6 family. Cancer Cell.

[96] Jones S.A., Jenkins B.J. (2018). Recent insights into targeting the IL-6 cytokine family in inflammatory diseases and cancer. Nat. Rev. Immunol..

[97] Lee Y.S., Choi I., Ning Y., Kim N.Y., Khatchadourian V., Yang D., Chung H.K., Choi D., LaBonte M.J., Ladner R.D. (2012). Interleukin-8 and its receptor CXCR2 in the tumour microenvironment promote colon cancer growth, progression and metastasis. Br. J. Cancer.

[98] Kern L., Mittenbuhler M.J., Vesting A.J., Ostermann A.L., Wunderlich C.M., Wunderlich F.T. (2018). Obesity-Induced TNFalpha and IL-6 Signaling: The Missing Link between Obesity and Inflammation-Driven Liver and Colorectal Cancers. Cancers.

[99] Najdaghi S., Razi S., Rezaei N. (2020). An overview of the role of interleukin-8 in colorectal cancer. Cytokine.

[100] O’Hara A.M., Bhattacharyya A., Bai J., Mifflin R.C., Ernst P.B., Mitra S., Crowe S.E. (2009). Tumor necrosis factor (TNF)-alpha-induced IL-8 expression in gastric epithelial cells: Role of reactive oxygen species and AP endonuclease-1/redox factor (Ref)-1. Cytokine.

[101] Amin M.N., Siddiqui S.A., Ibrahim M., Hakim M.L., Ahammed M.S., Kabir A., Sultana F. (2020). Inflammatory cytokines in the pathogenesis of cardiovascular disease and cancer. SAGE Open Med..

[102] Niess J.H., Hruz P., Kaymak T. (2018). The Interleukin-20 Cytokines in Intestinal Diseases. Front. Immunol..

[103] Kantola T., Klintrup K., Vayrynen J.P., Vornanen J., Bloigu R., Karhu T., Herzig K.H., Napankangas J., Makela J., Karttunen T.J. (2012). Stage-dependent alterations of the serum cytokine pattern in colorectal carcinoma. Br. J. Cancer.

[104] Kryczek I., Lin Y., Nagarsheth N., Peng D., Zhao L., Zhao E., Vatan L., Szeliga W., Dou Y., Owens S. (2014). IL-22(+)CD4(+) T cells promote colorectal cancer stemness via STAT3 transcription factor activation and induction of the methyltransferase DOT1L. Immunity.

[105] Gronke K., Hernandez P.P., Zimmermann J., Klose C.S.N., Kofoed-Branzk M., Guendel F., Witkowski M., Tizian C., Amann L., Schumacher F. (2019). Triantafyllopoulou, A.; Diefenbach, A. Interleukin-22 protects intestinal stem cells against genotoxic stress. Nature.

[106] Hue S., Ahern P., Buonocore S., Kullberg M.C., Cua D.J., McKenzie B.S., Powrie F., Maloy K.J. (2006). Interleukin-23 drives innate and T cell-mediated intestinal inflammation. J. Exp. Med..

[107] Yen D., Cheung J., Scheerens H., Poulet F., McClanahan T., McKenzie B., Kleinschek M.A., Owyang A., Mattson J., Blumenschein W. (2006). IL-23 is essential for T cell-mediated colitis and promotes inflammation via IL-17 and IL-6. J. Clin. Investig..

[108] Teymouri M., Pirro M., Fallarino F., Gargaro M., Sahebkar A. (2018). IL-35, a hallmark of immune-regulation in cancer progression, chronic infections and inflammatory diseases. Int. J. Cancer.

[109] Grivennikov S.I., Wang K., Mucida D., Stewart C.A., Schnabl B., Jauch D., Taniguchi K., Yu G.Y., Osterreicher C.H., Hung K.E. (2012). Adenoma-linked barrier defects and microbial products drive IL-23/IL-17-mediated tumour growth. Nature.

[110] Zepp J.A., Zhao J., Liu C., Bulek K., Wu L., Chen X., Hao Y., Wang Z., Wang X., Ouyang W. (2017). IL-17A-Induced PLET1 Expression Contributes to Tissue Repair and Colon Tumorigenesis. J. Immunol..

[111] Chung A.S., Wu X., Zhuang G., Ngu H., Kasman I., Zhang J., Vernes J.M., Jiang Z., Meng Y.G., Peale F.V. (2013). An interleukin-17-mediated paracrine network promotes tumor resistance to anti-angiogenic therapy. Nat. Med..

[112] Tosolini M., Kirilovsky A., Mlecnik B., Fredriksen T., Mauger S., Bindea G., Berger A., Bruneval P., Fridman W.H., Pages F. (2011). Clinical impact of different classes of infiltrating T cytotoxic and helper cells (Th1, th2, treg, th17) in patients with colorectal cancer. Cancer Res..

[113] Richmond J., Tuzova M., Cruikshank W., Center D. (2014). Regulation of cellular processes by interleukin-16 in homeostasis and cancer. J. Cell Physiol..

[114] Baghdadi M., Umeyama Y., Hama N., Kobayashi T., Han N., Wada H., Seino K.I. (2018). Interleukin-34, a comprehensive review. J. Leukoc. Biol..

[115] Franze E., Di Grazia A., Sica G.S., Biancone L., Laudisi F., Monteleone G. (2020). Interleukin-34 Enhances the Tumor Promoting Function of Colorectal Cancer-Associated Fibroblasts. Cancers.

[116] Franze E., Stolfi C., Troncone E., Scarozza P., Monteleone G. (2020). Role of Interleukin-34 in Cancer. Cancers.

[117] Kobelt D., Zhang C., Clayton-Lucey I.A., Glauben R., Voss C., Siegmund B., Stein U. (2020). Pro-inflammatory TNF-alpha and IFN-gamma Promote Tumor Growth and Metastasis via Induction of MACC1. Front. Immunol..

[118] Balkwill F. (2009). Tumour necrosis factor and cancer. Nat. Rev. Cancer.

[119] Popivanova B.K., Kitamura K., Wu Y., Kondo T., Kagaya T., Kaneko S., Oshima M., Fujii C., Mukaida N. (2008). Blocking TNF-alpha in mice reduces colorectal carcinogenesis associated with chronic colitis. J. Clin. Investig..

[120] Ogawa R., Yamamoto T., Hirai H., Hanada K., Kiyasu Y., Nishikawa G., Mizuno R., Inamoto S., Itatani Y., Sakai Y. (2019). Loss of SMAD4 Promotes Colorectal Cancer Progression by Recruiting Tumor-Associated Neutrophils via the CXCL1/8-CXCR2 Axis. Clin. Cancer Res..

[121] Xia C., He L., Sun Y. (2022). Expression and Prognostic Role of CXCL1 Gene in Colorectal Adenocarcinoma. Comput. Intell. Neurosci..

[122] Chen M.C., Baskaran R., Lee N.H., Hsu H.H., Ho T.J., Tu C.C., Lin Y.M., Viswanadha V.P., Kuo W.W., Huang C.Y. (2019). CXCL2/CXCR2 axis induces cancer stem cell characteristics in CPT-11-resistant LoVo colon cancer cells via Galphai-2 and Galphaq/11. J. Cell Physiol..

[123] Teijeira A., Garasa S., Gato M., Alfaro C., Migueliz I., Cirella A., de Andrea C., Ochoa M.C., Otano I., Etxeberria I. (2020). CXCR1 and CXCR2 Chemokine Receptor Agonists Produced by Tumors Induce Neutrophil Extracellular Traps that Interfere with Immune Cytotoxicity. Immunity.

[124] Fisher R.C., Bellamkonda K., Alex Molina L., Xiang S., Liska D., Sarvestani S.K., Chakrabarti S., Berg A., Jorgensen M.L., Hatala D. (2019). Disrupting Inflammation-Associated CXCL8-CXCR1 Signaling Inhibits Tumorigenicity Initiated by Sporadic- and Colitis-Colon Cancer Stem Cells. Neoplasia.

[125] Pennel K.A., Quinn J.A., Nixon C., Inthagard J., van Wyk H.C., Chang D., Rebus S., Group G., Hay J., Maka N.N. (2022). CXCL8 expression is associated with advanced stage, right sidedness, and distinct histological features of colorectal cancer. J Pathol. Clin. Res..

[126] Biasci D., Smoragiewicz M., Connell C.M., Wang Z., Gao Y., Thaventhiran J.E.D., Basu B., Magiera L., Johnson T.I., Bax L. (2020). CXCR4 inhibition in human pancreatic and colorectal cancers induces an integrated immune response. Proc. Natl. Acad. Sci. USA.

[127] Karin M., Shalapour S. (2022). Regulation of antitumor immunity by inflammation-induced epigenetic alterations. Cell Mol. Immunol..

[128] Wang W., Ding C.L., Wu M.X., Guo W., Hu R., Liu Y., Qi Z.T., Jia X.M. (2022). RAI16 maintains intestinal homeostasis and inhibits NLRP3-dependent IL-18/CXCL16-induced colitis and the progression of colitis-associated colorectal cancer. Clin. Transl. Med..

[129] Hao Q., Vadgama J.V., Wang P. (2020). CCL2/CCR2 signaling in cancer pathogenesis. Cell Commun. Signal..

[130] Xu M., Wang Y., Xia R., Wei Y., Wei X. (2021). Role of the CCL2-CCR2 signalling axis in cancer: Mechanisms and therapeutic targeting. Cell Prolif..

[131] Ma X., Su J., Zhao S., He Y., Li S., Yang X., Zhai S., Rong S., Zhang X., Xu G. (2022). CCL3 Promotes Proliferation of Colorectal Cancer Related with TRAF6/NF-kappaB Molecular Pathway. Contrast. Media Mol. Imaging.

[132] De la Fuente Lopez M., Landskron G., Parada D., Dubois-Camacho K., Simian D., Martinez M., Romero D., Roa J.C., Chahuan I., Gutierrez R. (2018). The relationship between chemokines CCL2, CCL3, and CCL4 with the tumor microenvironment and tumor-associated macrophage markers in colorectal cancer. Tumour Biol..

[133] Braoudaki M., Ahmad M.S., Mustafov D., Seriah S., Siddiqui M.N., Siddiqui S.S. (2022). Chemokines and chemokine receptors in colorectal cancer; multifarious roles and clinical impact. Semin. Cancer Biol..

[134] Strasly M., Doronzo G., Cappello P., Valdembri D., Arese M., Mitola S., Moore P., Alessandri G., Giovarelli M., Bussolino F. (2004). CCL16 activates an angiogenic program in vascular endothelial cells. Blood.

[135] Wagsater D., Dienus O., Lofgren S., Hugander A., Dimberg J. (2008). Quantification of the chemokines CCL17 and CCL22 in human colorectal adenocarcinomas. Mol. Med. Rep..

[136] Frick V.O., Rubie C., Keilholz U., Ghadjar P. (2016). Chemokine/chemokine receptor pair CCL20/CCR6 in human colorectal malignancy: An overview. World J. Gastroenterol..

[137] Vicinus B., Rubie C., Stegmaier N., Frick V.O., Kolsch K., Kauffels A., Ghadjar P., Wagner M., Glanemann M. (2013). miR-21 and its target gene CCL20 are both highly overexpressed in the microenvironment of colorectal tumors: Significance of their regulation. Oncol. Rep..

[138] Fung K.Y., Nguyen P.M., Putoczki T.L. (2020). Emerging Roles for Interleukin-18 in the Gastrointestinal Tumor Microenvironment. Adv. Exp. Med. Biol..

[139] Salcedo R., Worschech A., Cardone M., Jones Y., Gyulai Z., Dai R.M., Wang E., Ma W., Haines D., O’hUigin C. (2010). MyD88-mediated signaling prevents development of adenocarcinomas of the colon: Role of interleukin 18. J. Exp. Med..

[140] Weinstein A.M., Giraldo N.A., Petitprez F., Julie C., Lacroix L., Peschaud F., Emile J.F., Marisa L., Fridman W.H., Storkus W.J. (2019). Association of IL-36gamma with tertiary lymphoid structures and inflammatory immune infiltrates in human colorectal cancer. Cancer Immunol. Immunother..

[141] Elias M., Zhao S., Le H.T., Wang J., Neurath M.F., Neufert C., Fiocchi C., Rieder F. (2021). IL-36 in chronic inflammation and fibrosis—bridging the gap?. J. Clin. Investig..

[142] Liu H., Zheng R., Wang P., Yang H., He X., Ji Q., Bai W., Chen H., Chen J., Peng W. (2017). IL-37 Confers Protection against Mycobacterial Infection Involving Suppressing Inflammation and Modulating T Cell Activation. PLoS ONE.

[143] Dimberg J., Shamoun L., Landerholm K., Andersson R.E., Kolodziej B., Wagsater D. (2019). Genetic Variants of the IL2 Gene Related to Risk and Survival in Patients With Colorectal Cancer. Anticancer Res..

[144] Morre M., Beq S. (2012). Interleukin-7 and immune reconstitution in cancer patients: A new paradigm for dramatically increasing overall survival. Target Oncol..

[145] Gao J., Zhao L., Wan Y.Y., Zhu B. (2015). Mechanism of Action of IL-7 and Its Potential Applications and Limitations in Cancer Immunotherapy. Int. J. Mol. Sci..

[146] Wan J., Wu Y., Ji X., Huang L., Cai W., Su Z., Wang S., Xu H. (2020). IL-9 and IL-9-producing cells in tumor immunity. Cell Commun. Signal..

[147] Wang J., Sun M., Zhao H., Huang Y., Li D., Mao D., Zhang Z., Zhu X., Dong X., Zhao X. (2019). IL-9 Exerts Antitumor Effects in Colon Cancer and Transforms the Tumor Microenvironment In Vivo. Technol. Cancer Res. Treat..

[148] Mishra A., Sullivan L., Caligiuri M.A. (2014). Molecular pathways: Interleukin-15 signaling in health and in cancer. Clin. Cancer Res..

[149] Jabri B., Abadie V. (2015). IL-15 functions as a danger signal to regulate tissue-resident T cells and tissue destruction. Nat. Rev. Immunol..

[150] Ciszewski C., Discepolo V., Pacis A., Doerr N., Tastet O., Mayassi T., Maglio M., Basheer A., Al-Mawsawi L.Q., Green P.H.R. (2020). Identification of a gammac Receptor Antagonist That Prevents Reprogramming of Human Tissue-resident Cytotoxic T Cells by IL15 and IL21. Gastroenterology.

[151] Ong C.Y., Abdalkareem E.A., Khoo B.Y. (2022). Functional roles of cytokines in infectious disease associated colorectal carcinogenesis. Mol. Biol. Rep..

[152] Li Y., Cong Y., Jia M., He Q., Zhong H., Zhao Y., Li H., Yan M., You J., Liu J. (2021). Targeting IL-21 to tumor-reactive T cells enhances memory T cell responses and anti-PD-1 antibody therapy. Nat. Commun..

[153] Seo H., Jeon I., Kim B.S., Park M., Bae E.A., Song B., Koh C.H., Shin K.S., Kim I.K., Choi K. (2017). IL-21-mediated reversal of NK cell exhaustion facilitates anti-tumour immunity in MHC class I-deficient tumours. Nat. Commun..

[154] Menezes M.E., Bhatia S., Bhoopathi P., Das S.K., Emdad L., Dasgupta S., Dent P., Wang X.Y., Sarkar D., Fisher P.B. (2014). MDA-7/IL-24: Multifunctional cancer killing cytokine. Adv. Exp. Med. Biol..

[155] Malvicini M., Rizzo M., Alaniz L., Pinero F., Garcia M., Atorrasagasti C., Aquino J.B., Rozados V., Scharovsky O.G., Matar P. (2009). A novel synergistic combination of cyclophosphamide and gene transfer of interleukin-12 eradicates colorectal carcinoma in mice. Clin. Cancer Res.

[156] Yan J., Smyth M.J., Teng M.W.L. (2018). Interleukin (IL)-12 and IL-23 and Their Conflicting Roles in Cancer. Cold Spring Harb. Perspect. Biol..

[157] Razi S., Baradaran Noveiry B., Keshavarz-Fathi M., Rezaei N. (2019). IL-17 and colorectal cancer: From carcinogenesis to treatment. Cytokine.

[158] Zhang C., Hou D., Wei H., Zhao M., Yang L., Liu Q., Zhang X., Gong Y., Shao C. (2016). Lack of interferon-gamma receptor results in a microenvironment favorable for intestinal tumorigenesis. Oncotarget.

[159] Simpson J.A., Al-Attar A., Watson N.F., Scholefield J.H., Ilyas M., Durrant L.G. (2010). Intratumoral T cell infiltration, MHC class I and STAT1 as biomarkers of good prognosis in colorectal cancer. Gut.

[160] Mowat C., Mosley S.R., Namdar A., Schiller D., Baker K. (2021). Anti-tumor immunity in mismatch repair-deficient colorectal cancers requires type I IFN-driven CCL5 and CXCL10. J. Exp. Med..

[161] Markowitz S., Wang J., Myeroff L., Parsons R., Sun L., Lutterbaugh J., Fan R.S., Zborowska E., Kinzler K.W., Vogelstein B. (1995). Inactivation of the type II TGF-beta receptor in colon cancer cells with microsatellite instability. Science.

[162] Tauriello D.V.F., Sancho E., Batlle E. (2022). Overcoming TGFbeta-mediated immune evasion in cancer. Nat. Rev. Cancer.

[163] Bergamaschi C., Pandit H., Nagy B.A., Stellas D., Jensen S.M., Bear J., Cam M., Valentin A., Fox B.A., Felber B.K. (2020). Heterodimeric IL-15 delays tumor growth and promotes intratumoral CTL and dendritic cell accumulation by a cytokine network involving XCL1, IFN-gamma, CXCL9 and CXCL10. J. Immunother. Cancer.

[164] Akeus P., Szeponik L., Ahlmanner F., Sundstrom P., Alsen S., Gustavsson B., Sparwasser T., Raghavan S., Quiding-Jarbrink M. (2018). Regulatory T cells control endothelial chemokine production and migration of T cells into intestinal tumors of APC(min/+) mice. Cancer Immunol. Immunother..

[165] Shang S., Yang Y.W., Chen F., Yu L., Shen S.H., Li K., Cui B., Lv X.X., Zhang C., Yang C. (2022). TRIB3 reduces CD8(+) T cell infiltration and induces immune evasion by repressing the STAT1-CXCL10 axis in colorectal cancer. Sci. Transl. Med..

[166] Cao Y., Jiao N., Sun T., Ma Y., Zhang X., Chen H., Hong J., Zhang Y. (2021). CXCL11 Correlates With Antitumor Immunity and an Improved Prognosis in Colon Cancer. Front. Cell Dev. Biol..

[167] Zhang Y., Davis C., Shah S., Hughes D., Ryan J.C., Altomare D., Pena M.M. (2017). IL-33 promotes growth and liver metastasis of colorectal cancer in mice by remodeling the tumor microenvironment and inducing angiogenesis. Mol. Carcinog..

[168] Zhou Y., Ji Y., Wang H., Zhang H., Zhou H. (2018). IL-33 Promotes the Development of Colorectal Cancer Through Inducing Tumor-Infiltrating ST2L(+) Regulatory T Cells in Mice. Technol. Cancer Res. Treat.

[169] Luo P., Deng S., Ye H., Yu X., Deng Q., Zhang Y., Jiang L., Li J., Yu Y., Han W. (2020). The IL-33/ST2 pathway suppresses murine colon cancer growth and metastasis by upregulating CD40 L signaling. Biomed. Pharmacother..

[170] Eissmann M.F., Dijkstra C., Wouters M.A., Baloyan D., Mouradov D., Nguyen P.M., Davalos-Salas M., Putoczki T.L., Sieber O.M., Mariadason J.M. (2018). Interleukin 33 Signaling Restrains Sporadic Colon Cancer in an Interferon-gamma-Dependent Manner. Cancer Immunol. Res..

[171] Cui G. (2019). TH9, TH17, and TH22 Cell Subsets and Their Main Cytokine Products in the Pathogenesis of Colorectal Cancer. Front. Oncol..

[172] Borowczak J., Szczerbowski K., Maniewski M., Kowalewski A., Janiczek-Polewska M., Szylberg A., Marszalek A., Szylberg L. (2022). The Role of Inflammatory Cytokines in the Pathogenesis of Colorectal Carcinoma-Recent Findings and Review. Biomedicines.

[173] Deng S., Deng Q., Zhang Y., Ye H., Yu X., Zhang Y., Han G.Y., Luo P., Wu M., Yu Y. (2019). Non-platelet-derived CXCL4 differentially regulates cytotoxic and regulatory T cells through CXCR3 to suppress the immune response to colon cancer. Cancer Lett..

[174] Gao L.F., Zhong Y., Long T., Wang X., Zhu J.X., Wang X.Y., Hu Z.Y., Li Z.G. (2022). Tumor bud-derived CCL5 recruits fibroblasts and promotes colorectal cancer progression via CCR5-SLC25A24 signaling. J. Exp. Clin. Cancer Res..

[175] Suenaga M., Zhang W.U., Mashima T., Schirripa M., Cao S., Okazaki S., Berger M.D., Miyamoto Y., Barzi A., Yamaguchi T. (2021). Potential Molecular Cross Talk Among CCR5 Pathway Predicts Regorafenib Responsiveness in Metastatic Colorectal Cancer Patients. Cancer Genom. Proteom..

[176] Levy M., Blacher E., Elinav E. (2017). Microbiome, metabolites and host immunity. Curr. Opin. Microbiol..

[177] McBurney M.I., Davis C., Fraser C.M., Schneeman B.O., Huttenhower C., Verbeke K., Walter J., Latulippe M.E. (2019). Establishing What Constitutes a Healthy Human Gut Microbiome: State of the Science, Regulatory Considerations, and Future Directions. J. Nutr..

[178] Maukonen J., Saarela M. (2015). Human gut microbiota: Does diet matter?. Proc. Nutr. Soc..

[179] Kostic A.D., Gevers D., Pedamallu C.S., Michaud M., Duke F., Earl A.M., Ojesina A.I., Jung J., Bass A.J., Tabernero J. (2012). Genomic analysis identifies association of Fusobacterium with colorectal carcinoma. Genome Res..

[180] Khalyfa A.A., Punatar S., Aslam R., Yarbrough A. (2021). Exploring the Inflammatory Pathogenesis of Colorectal Cancer. Diseases.

[181] Hanus M., Parada-Venegas D., Landskron G., Wielandt A.M., Hurtado C., Alvarez K., Hermoso M.A., Lopez-Kostner F., De la Fuente M. (2021). Immune System, Microbiota, and Microbial Metabolites: The Unresolved Triad in Colorectal Cancer Microenvironment. Front. Immunol..

[182] Solis A.G., Klapholz M., Zhao J., Levy M. (2020). The bidirectional nature of microbiome-epithelial cell interactions. Curr. Opin. Microbiol..

[183] Nastasi C., Candela M., Bonefeld C.M., Geisler C., Hansen M., Krejsgaard T., Biagi E., Andersen M.H., Brigidi P., Odum N. (2015). The effect of short-chain fatty acids on human monocyte-derived dendritic cells. Sci. Rep..

[184] Rubinstein M.R., Wang X., Liu W., Hao Y., Cai G., Han Y.W. (2013). *Fusobacterium nucleatum* promotes colorectal carcinogenesis by modulating E-cadherin/beta-catenin signaling via its FadA adhesin. Cell Host Microbe.

[185] Boleij A., Hechenbleikner E.M., Goodwin A.C., Badani R., Stein E.M., Lazarev M.G., Ellis B., Carroll K.C., Albesiano E., Wick E.C. (2015). The Bacteroides fragilis toxin gene is prevalent in the colon mucosa of colorectal cancer patients. Clin. Infect. Dis..

[186] Wilson M.R., Jiang Y., Villalta P.W., Stornetta A., Boudreau P.D., Carra A., Brennan C.A., Chun E., Ngo L., Samson L.D. (2019). The human gut bacterial genotoxin colibactin alkylates DNA. Science.

[187] Atreya I., Neurath M.F. (2022). How the Tumor Micromilieu Modulates the Recruitment and Activation of Colorectal Cancer-Infiltrating Lymphocytes. Biomedicines..

[188] Chinnaiyan A.M., Tepper C.G., Seldin M.F., O’Rourke K., Kischkel F.C., Hellbardt S., Krammer P.H., Peter M.E., Dixit V.M. (1996). FADD/MORT1 is a common mediator of CD95 (Fas/APO-1) and tumor necrosis factor receptor-induced apoptosis. J. Biol. Chem..

[189] Lin Y., He Z., Ye J., Liu Z., She X., Gao X., Liang R. (2020). Progress in Understanding the IL-6/STAT3 Pathway in Colorectal Cancer. OncoTargets Ther..

[190] McFarlane A.J., Fercoq F., Coffelt S.B., Carlin L.M. (2021). Neutrophil dynamics in the tumor microenvironment. J. Clin. Investig..

[191] Shaul M.E., Fridlender Z.G. (2021). The dual role of neutrophils in cancer. Semin. Immunol..

[192] Andzinski L., Kasnitz N., Stahnke S., Wu C.F., Gereke M., von Kockritz-Blickwede M., Schilling B., Brandau S., Weiss S., Jablonska J. (2016). Type I IFNs induce anti-tumor polarization of tumor associated neutrophils in mice and human. Int. J. Cancer.

[193] Sahai E., Astsaturov I., Cukierman E., DeNardo D.G., Egeblad M., Evans R.M., Fearon D., Greten F.R., Hingorani S.R., Hunter T. (2020). A framework for advancing our understanding of cancer-associated fibroblasts. Nat. Rev. Cancer.

[194] Nicolas A.M., Pesic M., Engel E., Ziegler P.K., Diefenhardt M., Kennel K.B., Buettner F., Conche C., Petrocelli V., Elwakeel E. (2022). Inflammatory fibroblasts mediate resistance to neoadjuvant therapy in rectal cancer. Cancer Cell.

[195] Murai M., Turovskaya O., Kim G., Madan R., Karp C.L., Cheroutre H., Kronenberg M. (2009). Interleukin 10 acts on regulatory T cells to maintain expression of the transcription factor Foxp3 and suppressive function in mice with colitis. Nat. Immunol..

[196] Ruder B., Becker C. (2020). At the Forefront of the Mucosal Barrier: The Role of Macrophages in the Intestine. Cells.

[197] Wang L., Wang Y., Song Z., Chu J., Qu X. (2015). Deficiency of interferon-gamma or its receptor promotes colorectal cancer development. J. Interferon. Cytokine Res..

[198] Zhong X., Chen B., Yang Z. (2018). The Role of Tumor-Associated Macrophages in Colorectal Carcinoma Progression. Cell Physiol. Biochem..

[199] Herbeuval J.P., Lelievre E., Lambert C., Dy M., Genin C. (2004). Recruitment of STAT3 for production of IL-10 by colon carcinoma cells induced by macrophage-derived IL-6. J. Immunol..

[200] Dufait I., Schwarze J.K., Liechtenstein T., Leonard W., Jiang H., Escors D., De Ridder M., Breckpot K. (2015). Ex vivo generation of myeloid-derived suppressor cells that model the tumor immunosuppressive environment in colorectal cancer. Oncotarget.

[201] Sasidharan Nair V., Saleh R., Taha R.Z., Toor S.M., Murshed K., Ahmed A.A., Kurer M.A., Abu Nada M., Al Ejeh F., Elkord E. (2020). Differential gene expression of tumor-infiltrating CD4(+) T cells in advanced versus early stage colorectal cancer and identification of a gene signature of poor prognosis. Oncoimmunology.

[202] Bai Z., Zhou Y., Ye Z., Xiong J., Lan H., Wang F. (2021). Tumor-Infiltrating Lymphocytes in Colorectal Cancer: The Fundamental Indication and Application on Immunotherapy. Front. Immunol..

[203] Kistner L., Doll D., Holtorf A., Nitsche U., Janssen K.P. (2017). Interferon-inducible CXC-chemokines are crucial immune modulators and survival predictors in colorectal cancer. Oncotarget.

[204] Liu S.S., Yang Y.Z., Jiang C., Quan Q., Xie Q.K., Wang X.P., He W.Z., Rong Y.M., Chen P., Yang Q. (2018). Comparison of immunological characteristics between paired mismatch repair-proficient and -deficient colorectal cancer patients. J. Transl. Med..

[205] Sallusto F. (2016). Heterogeneity of Human CD4(+) T Cells Against Microbes. Annu. Rev. Immunol..

[206] Bule P., Aguiar S.I., Aires-Da-Silva F., Dias J.N.R. (2021). Chemokine-Directed Tumor Microenvironment Modulation in Cancer Immunotherapy. Int. J. Mol. Sci..

[207] Koller F.L., Hwang D.G., Dozier E.A., Fingleton B. (2010). Epithelial interleukin-4 receptor expression promotes colon tumor growth. Carcinogenesis.

[208] Gerlach K., Hwang Y., Nikolaev A., Atreya R., Dornhoff H., Steiner S., Lehr H.A., Wirtz S., Vieth M., Waisman A. (2014). TH9 cells that express the transcription factor PU.1 drive T cell-mediated colitis via IL-9 receptor signaling in intestinal epithelial cells. Nat. Immunol..

[209] Huang Y., Cao Y., Zhang S., Gao F. (2015). Association between low expression levels of interleukin-9 and colon cancer progression. Exp. Ther. Med..

[210] De Simone V., Franze E., Ronchetti G., Colantoni A., Fantini M.C., Di Fusco D., Sica G.S., Sileri P., MacDonald T.T., Pallone F. (2015). Th17-type cytokines, IL-6 and TNF-alpha synergistically activate STAT3 and NF-kB to promote colorectal cancer cell growth. Oncogene.

[211] Perez L.G., Kempski J., McGee H.M., Pelzcar P., Agalioti T., Giannou A., Konczalla L., Brockmann L., Wahib R., Xu H. (2020). TGF-beta signaling in Th17 cells promotes IL-22 production and colitis-associated colon cancer. Nat. Commun..

[212] Llosa N.J., Luber B., Tam A.J., Smith K.N., Siegel N., Awan A.H., Fan H., Oke T., Zhang J., Domingue J. (2019). Intratumoral Adaptive Immunosuppression and Type 17 Immunity in Mismatch Repair Proficient Colorectal Tumors. Clin. Cancer Res..

[213] Fantini M.C., Favale A., Onali S., Facciotti F. (2020). Tumor Infiltrating Regulatory T Cells in Sporadic and Colitis-Associated Colorectal Cancer: The Red Little Riding Hood and the Wolf. Int. J. Mol. Sci..

[214] Akkaya B., Shevach E.M. (2020). Regulatory T cells: Master thieves of the immune system. Cell Immunol..

[215] Akkaya B., Oya Y., Akkaya M., Al Souz J., Holstein A.H., Kamenyeva O., Kabat J., Matsumura R., Dorward D.W., Glass D.D. (2019). Regulatory T cells mediate specific suppression by depleting peptide-MHC class II from dendritic cells. Nat. Immunol..

[216] Salama P., Phillips M., Grieu F., Morris M., Zeps N., Joseph D., Platell C., Iacopetta B. (2009). Tumor-infiltrating FOXP3+ T regulatory cells show strong prognostic significance in colorectal cancer. J. Clin. Oncol..

[217] Argon A., Vardar E., Kebat T., Erdinc O., Erkan N. (2016). The Prognostic Significance of FoxP3+ T Cells and CD8+ T Cells in Colorectal Carcinomas. J. Environ. Pathol. Toxicol. Oncol..

[218] Reimers M.S., Engels C.C., Putter H., Morreau H., Liefers G.J., van de Velde C.J., Kuppen P.J. (2014). Prognostic value of HLA class I, HLA-E, HLA-G and Tregs in rectal cancer: A retrospective cohort study. BMC Cancer.

[219] Fionda C., Scarno G., Stabile H., Molfetta R., Di Censo C., Gismondi A., Paolini R., Sozzani S., Santoni A., Sciume G. (2022). NK Cells and Other Cytotoxic Innate Lymphocytes in Colorectal Cancer Progression and Metastasis. Int. J. Mol. Sci..

[220] Rosser E.C., Mauri C. (2015). Regulatory B cells: Origin, phenotype, and function. Immunity.

[221] Melcher C., Yu J., Duong V.H.H., Westphal K., Helmi Siasi Farimany N., Shaverskyi A., Zhao B., Strowig T., Glage S., Brand K. (2022). B cell-mediated regulatory mechanisms control tumor-promoting intestinal inflammation. Cell Rep..

[222] Shimabukuro-Vornhagen A., Schlosser H.A., Gryschok L., Malcher J., Wennhold K., Garcia-Marquez M., Herbold T., Neuhaus L.S., Becker H.J., Fiedler A. (2014). Characterization of tumor-associated B-cell subsets in patients with colorectal cancer. Oncotarget.

[223] Subtil B., Cambi A., Tauriello D.V.F., de Vries I.J.M. (2021). The Therapeutic Potential of Tackling Tumor-Induced Dendritic Cell Dysfunction in Colorectal Cancer. Front. Immunol..

[224] Zong J., Keskinov A.A., Shurin G.V., Shurin M.R. (2016). Tumor-derived factors modulating dendritic cell function. Cancer Immunol. Immunother..

[225] Chow M.T., Ozga A.J., Servis R.L., Frederick D.T., Lo J.A., Fisher D.E., Freeman G.J., Boland G.M., Luster A.D. (2019). Intratumoral Activity of the CXCR3 Chemokine System Is Required for the Efficacy of Anti-PD-1 Therapy. Immunity.

[226] Pelka K., Hofree M., Chen J.H., Sarkizova S., Pirl J.D., Jorgji V., Bejnood A., Dionne D., Ge W.H., Xu K.H. (2021). Spatially organized multicellular immune hubs in human colorectal cancer. Cell.

[227] Wang Q., Shen X., Chen G., Du J. (2022). Drug Resistance in Colorectal Cancer: From Mechanism to Clinic. Cancers.

[228] Shaked Y. (2019). The pro-tumorigenic host response to cancer therapies. Nat. Rev. Cancer.

[229] Wang Y.J., Fletcher R., Yu J., Zhang L. (2018). Immunogenic effects of chemotherapy-induced tumor cell death. Genes Dis..

[230] Karagiannis G.S., Condeelis J.S., Oktay M.H. (2019). Chemotherapy-Induced Metastasis: Molecular Mechanisms, Clinical Manifestations, Therapeutic Interventions. Cancer Res..

[231] Silva V.R., Santos L.S., Dias R.B., Quadros C.A., Bezerra D.P. (2021). Emerging agents that target signaling pathways to eradicate colorectal cancer stem cells. Cancer Commun..

[232] Zhao H., Wu L., Yan G., Chen Y., Zhou M., Wu Y., Li Y. (2021). Inflammation and tumor progression: Signaling pathways and targeted intervention. Signal. Transduct. Target Ther..

[233] Coffelt S.B., Wellenstein M.D., de Visser K.E. (2016). Neutrophils in cancer: Neutral no more. Nat. Rev. Cancer.

[234] Homann L., Rentschler M., Brenner E., Bohm K., Rocken M., Wieder T. (2022). IFN-gamma and TNF Induce Senescence and a Distinct Senescence-Associated Secretory Phenotype in Melanoma. Cells.

[235] Rentschler M., Braumuller H., Briquez P.S., Wieder T. (2022). Cytokine-Induced Senescence in the Tumor Microenvironment and Its Effects on Anti-Tumor Immune Responses. Cancers.

[236] Braumuller H., Mauerer B., Berlin C., Plundrich D., Marbach P., Cauchy P., Laessle C., Biesel E., Holzner P.A., Kesselring R. (2022). Senescent Tumor Cells in the Peritoneal Carcinomatosis Drive Immunosenescence in the Tumor Microenvironment. Front. Immunol..

[237] Rodriguez-Ruiz M.E., Vitale I., Harrington K.J., Melero I., Galluzzi L. (2020). Immunological impact of cell death signaling driven by radiation on the tumor microenvironment. Nat. Immunol..

[238] Chan Wah Hak C.M.L., Rullan A., Patin E.C., Pedersen M., Melcher A.A., Harrington K.J. (2022). Enhancing anti-tumour innate immunity by targeting the DNA damage response and pattern recognition receptors in combination with radiotherapy. Front. Oncol..

[239] Yamazaki T., Vanpouille-Box C., Demaria S., Galluzzi L. (2020). Immunogenic Cell Death Driven by Radiation-Impact on the Tumor Microenvironment. Cancer Treat. Res..

[240] Zhang X., Zhang H., Zhang J., Yang M., Zhu M., Yin Y., Fan X., Yu F. (2022). The paradoxical role of radiation-induced cGAS-STING signalling network in tumour immunity. Immunology.

[241] Chiang C.S., Fu S.Y., Wang S.C., Yu C.F., Chen F.H., Lin C.M., Hong J.H. (2012). Irradiation promotes an m2 macrophage phenotype in tumor hypoxia. Front. Oncol..

[242] Kroemer G., Galluzzi L., Kepp O., Zitvogel L. (2013). Immunogenic cell death in cancer therapy. Annu. Rev. Immunol..

[243] Krysko D.V., Garg A.D., Kaczmarek A., Krysko O., Agostinis P., Vandenabeele P. (2012). Immunogenic cell death and DAMPs in cancer therapy. Nat. Rev. Cancer.

[244] McLaughlin M., Patin E.C., Pedersen M., Wilkins A., Dillon M.T., Melcher A.A., Harrington K.J. (2020). Inflammatory microenvironment remodelling by tumour cells after radiotherapy. Nat. Rev. Cancer.

[245] Crucitti A., Corbi M., Tomaiuolo P.M., Fanali C., Mazzari A., Lucchetti D., Migaldi M., Sgambato A. (2015). Laparoscopic surgery for colorectal cancer is not associated with an increase in the circulating levels of several inflammation-related factors. Cancer Biol. Ther..

[246] Predina J., Eruslanov E., Judy B., Kapoor V., Cheng G., Wang L.C., Sun J., Moon E.K., Fridlender Z.G., Albelda S. (2013). Changes in the local tumor microenvironment in recurrent cancers may explain the failure of vaccines after surgery. Proc. Natl. Acad. Sci. USA.

[247] Tartter P.I., Steinberg B., Barron D.M., Martinelli G. (1987). The prognostic significance of natural killer cytotoxicity in patients with colorectal cancer. Arch. Surg..

[248] De Assis J.V., Coutinho L.A., Oyeyemi I.T., Oyeyemi O.T., Grenfell R. (2022). Diagnostic and therapeutic biomarkers in colorectal cancer: A review. Am. J. Cancer Res..

[249] Janney A., Powrie F., Mann E.H. (2020). Host-microbiota maladaptation in colorectal cancer. Nature.

[250] Percario R., Panaccio P., di Mola F.F., Grottola T., Di Sebastiano P. (2021). The Complex Network between Inflammation and Colorectal Cancer: A Systematic Review of the Literature. Cancers.

[251] Wieder T., Eigentler T., Brenner E., Rocken M. (2018). Immune checkpoint blockade therapy. J. Allergy Clin. Immunol..

[252] Rabin H., Adamson R.H., Neubauer R.H., Cicmanec J.L., Wallen W.C. (1976). Pilot studies with human interferon in Herpesvirus saimiri-induced lymphoma in owl monkeys. Cancer Res..

[253] Youn J.K., Hovanessian A.G., Riviere Y., Hue G., Lacour F. (1983). Enhancement of natural killer cell activity and 2-5A synthetase in mice treated with polyadenylic.polyuridylic acid. Cell Immunol..

[254] Lacour J., Lacour F., Spira A., Michelson M., Petit J.Y., Delage G., Sarrazin D., Contesso G., Viguier J. (1980). Adjuvant treatment with polyadenylic-polyuridylic acid (Polya.Polyu) in operable breast cancer. Lancet.

[255] Quesada J.R., Gutterman J.U., Hersh E.M. (1982). Clinical and immunological study of beta interferon by intramuscular route in patients with metastatic breast cancer. J. Interferon. Res..

[256] Chaplinski T., Laszlo J., Moore J., Silverman P. (1983). Phase II trial of lymphoblastoid interferon in metastatic colon carcinoma. Cancer Treat. Rep..

[257] Lienard D., Ewalenko P., Delmotte J.J., Renard N., Lejeune F.J. (1992). High-dose recombinant tumor necrosis factor alpha in combination with interferon gamma and melphalan in isolation perfusion of the limbs for melanoma and sarcoma. J. Clin. Oncol..

[258] Ruegg C., Yilmaz A., Bieler G., Bamat J., Chaubert P., Lejeune F.J. (1998). Evidence for the involvement of endothelial cell integrin alphaVbeta3 in the disruption of the tumor vasculature induced by TNF and IFN-gamma. Nat. Med..

[259] Braumuller H., Wieder T., Brenner E., Assmann S., Hahn M., Alkhaled M., Schilbach K., Essmann F., Kneilling M., Griessinger C. (2013). T-helper-1-cell cytokines drive cancer into senescence. Nature.

[260] Duggan M.C., Jochems C., Donahue R.N., Richards J., Karpa V., Foust E., Paul B., Brooks T., Tridandapani S., Olencki T. (2016). A phase I study of recombinant (r) vaccinia-CEA(6D)-TRICOM and rFowlpox-CEA(6D)-TRICOM vaccines with GM-CSF and IFN-alpha-2b in patients with CEA-expressing carcinomas. Cancer Immunol. Immunother..

[261] Pawelec G., Borowitz A., Krammer P.H., Wernet P. (1982). Constitutive interleukin 2 production by the JURKAT human leukemic T cell line. Eur. J. Immunol..

[262] Mazumder A., Rosenberg S.A. (1984). Successful immunotherapy of natural killer-resistant established pulmonary melanoma metastases by the intravenous adoptive transfer of syngeneic lymphocytes activated in vitro by interleukin 2. J. Exp. Med..

[263] Schmidt-Wolf I.G., Finke S., Trojaneck B., Denkena A., Lefterova P., Schwella N., Heuft H.G., Prange G., Korte M., Takeya M. (1999). Phase I clinical study applying autologous immunological effector cells transfected with the interleukin-2 gene in patients with metastatic renal cancer, colorectal cancer and lymphoma. Br. J. Cancer.

[264] Wittig B., Marten A., Dorbic T., Weineck S., Min H., Niemitz S., Trojaneck B., Flieger D., Kruopis S., Albers A. (2001). Therapeutic vaccination against metastatic carcinoma by expression-modulated and immunomodified autologous tumor cells: A first clinical phase I/II trial. Hum. Gene Ther..

[265] Caporale A., Brescia A., Galati G., Castelli M., Saputo S., Terrenato I., Cucina A., Liverani A., Gasparrini M., Ciardi A. (2007). Locoregional IL-2 therapy in the treatment of colon cancer. Cell-induced lesions of a murine model. Anticancer Res..

[266] Hutmacher C., Gonzalo Nunez N., Liuzzi A.R., Becher B., Neri D. (2019). Targeted Delivery of IL2 to the Tumor Stroma Potentiates the Action of Immune Checkpoint Inhibitors by Preferential Activation of NK and CD8(+) T Cells. Cancer Immunol. Res..

[267] Campian J.L., Ghosh S., Kapoor V., Yan R., Thotala S., Jash A., Hu T., Mahadevan A., Rifai K., Page L. (2022). Long-Acting Recombinant Human Interleukin-7, NT-I7, Increases Cytotoxic CD8 T Cells and Enhances Survival in Mouse Glioma Models. Clin. Cancer Res..

